# Integrated analysis and transcript abundance modelling of H3K4me3 and H3K27me3 in developing secondary xylem

**DOI:** 10.1038/s41598-017-03665-1

**Published:** 2017-06-13

**Authors:** Steven G. Hussey, Mattheus T. Loots, Karen van der Merwe, Eshchar Mizrachi, Alexander A. Myburg

**Affiliations:** 1 Department of Genetics, Forestry and Agricultural Biotechnology Institute (FABI), University of Pretoria, Private Bag X20, Pretoria, 0028 South Africa; 2 Department of Statistics, University of Pretoria, Private Bag X20, Pretoria, 0028 South Africa; 3 Centre for Bioinformatics and Computational Biology, Genomics Research Institute (GRI), University of Pretoria, Private Bag X20, Pretoria, 0028 South Africa

## Abstract

Despite the considerable contribution of xylem development (xylogenesis) to plant biomass accumulation, its epigenetic regulation is poorly understood. Furthermore, the relative contributions of histone modifications to transcriptional regulation is not well studied in plants. We investigated the biological relevance of H3K4me3 and H3K27me3 in secondary xylem development using ChIP-seq and their association with transcript levels among other histone modifications in woody and herbaceous models. In developing secondary xylem of the woody model *Eucalyptus grandis*, H3K4me3 and H3K27me3 genomic spans were distinctly associated with xylogenesis-related processes, with (late) lignification pathways enriched for putative bivalent domains, but not early secondary cell wall polysaccharide deposition. H3K27me3-occupied genes, of which 753 (~31%) are novel targets, were enriched for transcriptional regulation and flower development and had significant preferential expression in roots. Linear regression models of the ChIP-seq profiles predicted ~50% of transcript abundance measured with strand-specific RNA-seq, confirmed in a parallel analysis in Arabidopsis where integration of seven additional histone modifications each contributed smaller proportions of unique information to the predictive models. This study uncovers the biological importance of histone modification antagonism and genomic span in xylogenesis and quantifies for the first time the relative correlations of histone modifications with transcript abundance in plants.

## Introduction

Eukaryotic genomes are compartmentalised into distinct chromatin states that profoundly influence transcriptional activity in a cell-specific manner. Post-translational modifications of histone N-terminal regions influence the local chromatin structure by altering their association with nucleosomal DNA or by recruiting chromatin re-modelling complexes^1, 2^. While dozens of histone modifications (HMs) have been described in plants, yeast, human, worm and fly, their roles in development and the mechanisms for their establishment, maintenance and removal are poorly understood. HMs are generally reset during embryogenesis, after which they regulate distinct sets of genes in different tissues during development.

Xylogenesis (wood formation, or secondary growth) represents a strong and permanent carbon sink in plants and a commitment to a cell fate ending in cell death. During this developmental process, xylem initial cells produced by the meristematic vascular cambium undergo elongation, secondary cell wall (SCW) deposition, lignification and finally programmed cell death (in the case of fibres and vessels) in a narrow tissue layer within the stem^3, 4^. The process is likely to be extensively regulated at the chromatin level, as demonstrated for example through the regulation of lignification by histone H1.3 in *Eucalyptus* ^5^. The majority of plant epigenomic studies, however, have been based on whole organs or complex mixtures of tissues that provide limited resolution of cell- or tissue-specific epigenetic regulation during development. An understanding of the epigenetic regulation of xylogenesis is especially limited due to the frequent use of herbaceous models such as Arabidopsis *(Arabidopsis thaliana*) in studies to date. One exception includes the analysis of DNA methylation in several poplar tissues, revealing hundreds of methylation sites unique to developing xylem, some of which influence alternative splicing^6^.

Trimethylated lysine-4 of histone H3 (H3K4me3) and trimethylation of H3 lysine-27 (H3K27me3) comprise two major HMs associated with gene activation and repression, respectively, and occupy contrasting chromatin environments^7, 8^. H3K4 trimethylation is generally established at the 5′ region of transcribed genes by homologs of the yeast SET1 histone methyltransferase^9, 10^, reinforcing transcription by recruiting pre-initiation complex machinery^11^. In contrast, H3K27me3 is deposited via histone methyltransferases in the conserved Polycomb Repressive Complex 2 (PRC2), recruited through the recognition of Polycomb Response Elements (PREs) in animals where H2K27me3-modified regions are broad and typically span several genes^12^. In the case of plants, H3K27me3 distributions are generally gene-specific^13^. In some cases, long noncoding RNAs have been shown to recruit H3K27me3 to developmentally important genes such as the well-studied *FLOWERING LOCUS C* model (reviewed by Hepworth and Dean)^14^, while in others PRC2 components appear to recognize PRE motifs also found in animals^15^.

Insights into the relative importance, redundancy and complementarity of different epigenomic marks can be revealed by predictive modelling, which approximates the unknown underlying mechanisms (reviewed by Budden *et al*.)^16^. Since HMs serve as indicators of transcriptional activity and chromatin state, there has been considerable interest in quantifying to what extent gene transcript levels are reflected by HM enrichment, chromatin accessibility and transcription factor binding site data. In mammals, worm, fly and yeast, over 50% of variation in transcript levels across genes can be predicted from only a few HMs^17–19^. Inclusion of additional HM data may yield R^2^ values of 0.55 (worm) or even 0.71 (fly)^19^. However, model accuracy depends heavily on the modelling approach, cell line and number of epigenomic marks considered. Optimal modelling approaches and the relative importance of different HMs remain to be explored in plants.

Given the paucity of HM profiles with tissue-level resolution in plants and our poor knowledge of epigenetic regulation in xylogenesis, we investigated the possible biological roles of the two predominant but antagonist HMs, H3K4me3 and H3K27me3, in early secondary xylem development and their associations with observed transcript levels. Our woody model, *Eucalyptus grandis* (Myrtaceae), is of particular significance not only because of its high value as a global wood fibre crop, but also its extensive accumulation of tandem gene duplications (the highest known in any plant genome) over the last ~50 million years (Myr) ago, which remains poorly understood from an evolutionary perspective^20^. This study contributes over 750 previously unknown H3K27me3 targets, reveals new insights into tissue-specific plant epigenetic regulation and shows that ~55% of transcript abundance variation across genes can be predicted from multiple HM datasets in Arabidopsis.

## Results

### Genomic distributions of H3K4me3 and H3K27me3 in developing xylem

**Figure 1:**
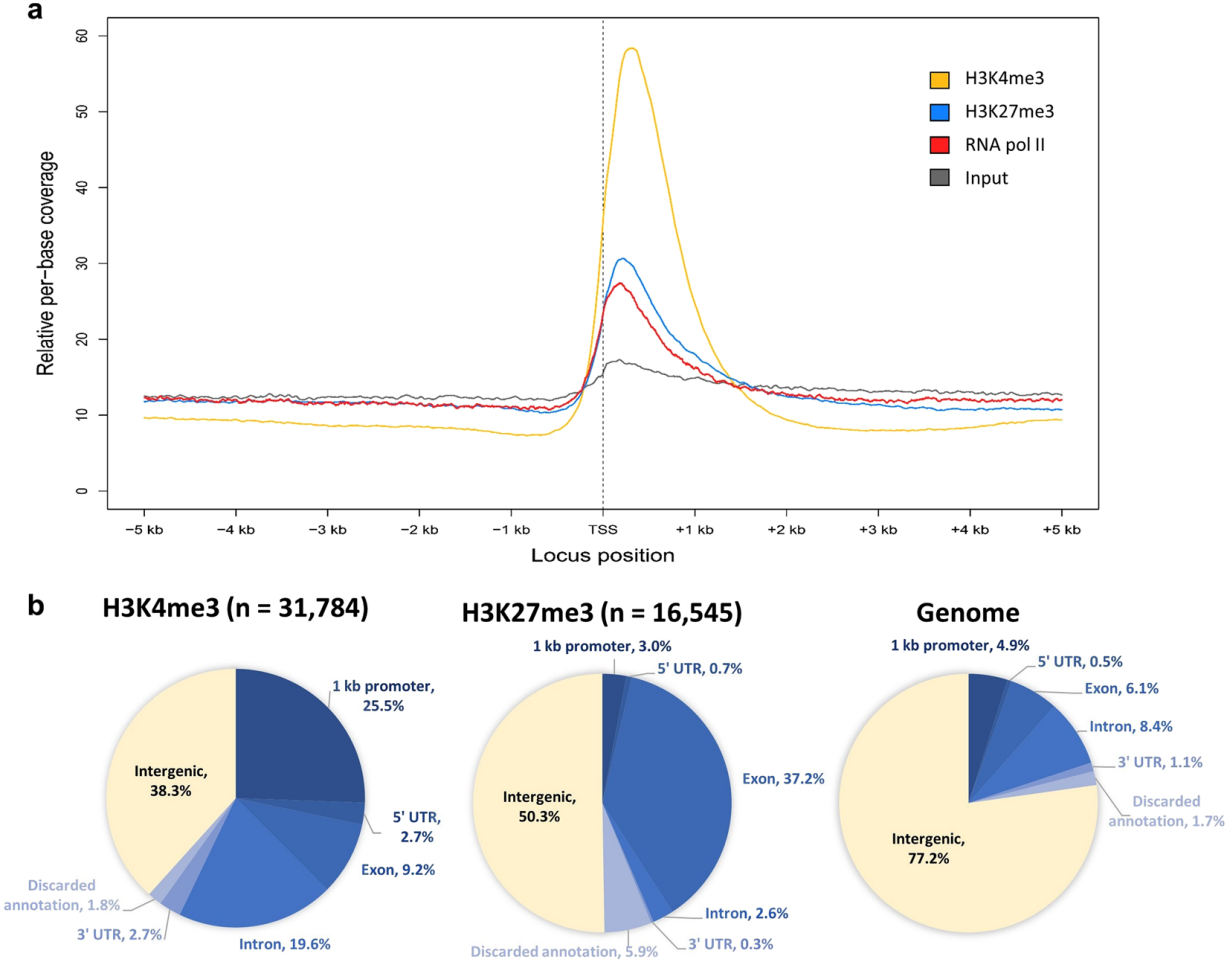
Genomic distribution of H3K4me3 and H3K27me3 in *E. grandis* developing xylem. (**a**) Relative per-base coverage of H3K4me3, H3K27me3, RNA pol II and input control libraries across annotated transcription start sites (TSS). (**b**) Genomic features coinciding with H3K4me3 and H3K27me3 ChIP-seq peak summits. The proportion of each annotation in the genome is indicated on the far right for comparison.

We previously described a genome-wide profile of H3K4me3 in the developing secondary xylem (DSX) tissues of field-grown *E. grandis* trees using chromatin immunoprecipitation sequencing (ChIP-seq)^21^. The ~1 mm DSX layer is exposed after peeling off the bark along the cambial layer, representing elongating xylem cells and early SCW deposition (Supplementary Fig. S1). We repeated the H3K4me3 ChIP-seq experiment at deeper coverage and also profiled H3K27me3 from the same tissue samples. After confirming the specificity of commercial ChIP-seq antibodies against H3K4me3 and H3K27me3 peptides (Supplementary Fig. S2), ChIP-seq was performed based on Encyclopedia of DNA Element (ENCODE) guidelines^22^, yielding highly immuno-enriched ChIP-seq libraries (Supplementary Fig. S3) with a low percentage of reads mapping to plastids (Supplementary Table S1). We determined the deposition preferences of H3K4me3 and H3K27me3 relative to transcriptional start sites (TSS) by plotting relative per-base library coverage across all annotated genes anchored at the TSS. H3K4me3 coverage peaked ~500 bp after the TSS as found previously^21^, while H3K27me3 peaked within 300 bp after the TSS (Fig. 1a). As a positive control for the TSS region, we plotted low-coverage RNA pol II ChIP-seq data derived from the same samples, revealing prominent enrichment over the input control across the TSS (Fig. 1a).

We identified 31,784 H3K4me3 and 16,545 H3K27me3 biologically reproducible ChIP-seq peaks (irreproducible discovery rate <0.01), covering ~5.1% and 2.7% of the genome, respectively (Supplementary Figs S4–S6, Supplementary Datasets 1 and 2), representing genomic regions where these HMs are deposited. Approx. 97% of the H3K4me3 peaks identified in our previous study^21^ were reproduced here. For H3K4me3, and to some extent H3K27me3, the peak summits were strongly biased towards genic features relative to the genome annotation (Fig. 1b). H3K4me3 peak density correlated with gene density (Pearson’s r = 0.88) but not with repetitive sequences (r = −0.03), while H3K27me3 peak density correlated fairly strongly with both genes (r = 0.48) and repetitive sequences (r = 0.31) (Supplementary Fig. S7).

Based on their physical overlap with ChIP-seq peaks, 19,605 and 5,776 genes were identified as occupied by H3K4me3 and H3K27me3, respectively (Supplementary Datasets 1 and 2). Of the H3K27me3 dataset, 2,708 genes (46.9%) were also occupied by H3K4me3 which may potentially indicate genes in a “bivalent” epigenomic state. This category was significantly smaller than expected (Fisher’s exact test, *P* = 3.3 × 10^−8^) despite a mildly positive correlation (r = 0.26) between the average nett signals of each modification was. We observed that tandemly duplicated genes, which encompass 34% of known genes in *E. grandis* ^20^, were enriched for H3K27me3 (1.64-fold, *P* ≈ 0, hypergeometric test) but depleted for H3K4me3 (1.42-fold, *P* ≈ 0), consistent with a report that TFL2/LHP1, the “reader” of H3K27me3, is enriched among tandemly duplicated genes in Arabidopsis^23^. We also investigated the association between the modified histones and transposable elements in *E. grandis* ^20^. Consistent with findings in Arabidopsis^24^, most retrotransposon (Class I) and DNA transposon (Class II) families were significantly depleted of H3K4me3 and H3K27me3 compared to a permuted null model (Supplementary Table S2). However, maverick/polinton type DNA transposons^25^ were highly enriched for H3K27me3. Since H3K27me3 targeting of transposable elements is highly tissue-specific^26^, it is unclear whether this enrichment is a general feature of *E. grandis* or a result specific to developing xylem.

### The genomic span of H3K4me3 and H3K27me3 deposition is related to distinct biological functions in developing xylem

Since the biology of H3K4me3 in DSX was investigated previously by our lab^21^, we explored for comparison the biological roles of H3K27-trimethylated genes in DSX. Given that H3K27me3 targets in secondary xylem tissue are unknown in plants, and to identify unique H3K27me3 targets in *E. grandis* DSX tissue, the closest Arabidopsis homologs of the 5,776 H3K27me3 targets in *E. grandis* were compared to an extensive set of published Arabidopsis H3K27me3 datasets comprised of seedlings, leaf, shoot apical meristem, callus, endosperm and root vascular cylinder^27–36^. Overall, ~69% of homologs of *E. grandis* H3K27-trimethylated genes were identified in previous studies (Supplementary Table S3), implying that at least 31% of the genes are previously unknown H3K27-trimethylated targets. Among these, the most significant of five overrepresented biological processes was that of response to hypoxia/oxygen levels (adj. *P* = 0.012), a process frequently associated with waterlogging-prone root development^37, 38^. Among all gene targets, transcription factor activity, transcriptional regulation and DNA-binding activity were highly enriched among H3K27me3-marked genes, as well as secondary metabolism and response to endogenous stimuli (Table 1). This contrasted markedly with the “housekeeping” functions overrepresented among H3K4-trimethylated genes, most of which were significantly underrepresented among H3K27-trimethylated genes (Table 1). Interestingly, flower development was significantly enriched among H3K27-trimethylated genes, represented by homologs of the well-studied TFs *AG*, *AP2*, *FLC*, *LFY*, *PI* and *SEP*, among others.

**Table 1:** GOSlim plant gene ontologies overrepresented among H3K4me3- and H3K27me3-modified genes.

Dataset	GO I.D.	Ontology description	*P*-value*	Enrichment
H3K4me3 (n = 19,605)	5737	Cytoplasm†	7.40e-33	1.08
	5622	Intracellular†	2.41e-29	1.06
	9536	Plastid†	1.12e-12	1.07
	5739	Mitochondrion†	8.37e-11	1.12
	5198	Structural molecule activity†	1.40e-09	1.16
	6412	Translation†	1.97e-09	1.15
	9058	Biosynthetic process†	1.61e-08	1.07
	5886	Plasma membrane	2.70e-05	1.06
	5840	Ribosome†	2.77e-05	1.15
	6139	Nucleobase, nucleoside, nucleotide and nucleic acid metabolic process†	8.28e-05	1.07
	9987	Cellular process†	1.21e-04	1.02
	19538	Protein metabolic process†	1.76e-04	1.04
	8152	Metabolic process†	3.39e-04	1.02
	3723	RNA binding†	3.39e-04	1.10
	5829	Cytosol†	4.55e-04	1.09
	9790	Embryonic development†	5.04e-04	1.09
	5794	Golgi apparatus†	3.65e-03	1.11
	8135	Translation factor activity, nucleic acid binding†	2.16e-02	1.13
	5634	Nucleus	2.61e-02	1.03
	5783	Endoplasmic reticulum	4.00e-02	1.07
H3K27me3 (n = 5,776)	3700	Transcription factor activity	3.99e-74	2.55
	30528	Transcription regulator activity	1.02e-60	2.28
	19825	Oxygen binding	2.26e-35	4.29
	3677	DNA binding	1.15e-32	1.78
	19748	Secondary metabolic process	3.75e-18	2.58
	9719	Response to endogenous stimulus	1.02e-15	1.84
	5215	Transporter activity	8.66e-07	1.44
	9607	Response to biotic stimulus	1.12e-06	1.64
	5576	Extracellular region	2.17e-06	1.87
	3676	Nucleic acid binding	3.47e-06	1.24
	30312	External encapsulating structure	1.52e-05	1.68
	5618	Cell wall	3.50e-05	1.65
	6519	Cellular amino acid and derivative metabolic process	4.00e-05	1.58
	3824	Catalytic activity	5.75e-05	1.11
	6950	Response to stress	9.98e-05	1.27
	7275	Multicellular organismal development	2.91e-04	1.25
	9908	Flower development	3.62e-04	1.74
	6629	Lipid metabolic process	1.60e-03	1.40
	30154	Cell differentiation	4.78e-03	1.52
	9653	Anatomical structure morphogenesis	1.24e-02	1.34
	6810	Transport	1.72e-02	1.19
	5634	Nucleus	1.80e-02	1.15
	5575	Cellular component	1.89e-02	1.03
	9628	Response to abiotic stimulus	2.24e-02	1.20

*Benjamini & Hochberg-corrected value.

^†^GO terms that were underrepresented among H3K27-trimethylated genes.

**Figure 2:**
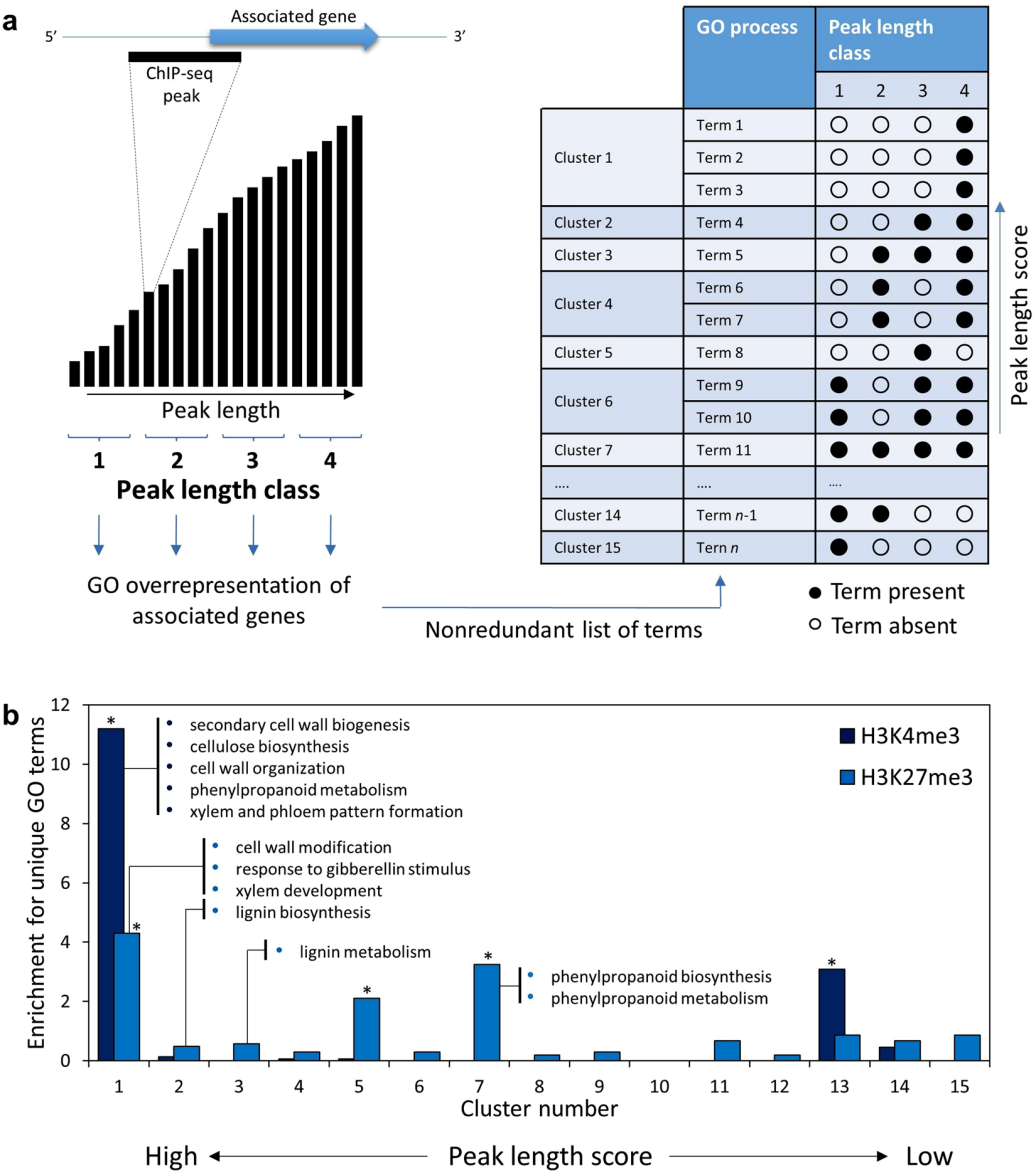
Enrichment of H3K4me3 and H3K27me3 peak length clusters for unique biological processes. (**a**) Schematic representation of method. (**b**) Degree of enrichment for unique biological processes for each cluster. Selected terms associated with xylogenesis are indicated for the clusters where they occur. *Significantly enriched above expectation; binomial test, *P* < 0.01.

The enrichment profile of a given HM (i.e. the genomic span and shape of a ChIP-seq peak, which reflect the number of successive nucleosomes at a locus and the proportion of nucleosomes at a given nucleosome position that are modified by a HM in a cell population, respectively) is related to the expression and biological functions of the associated genes^39, 40^. To investigate the relationship between biological functions and the span of HMs observed in xylem, we analysed the representation and distribution of biological functions among genes occupied by HMs of differing length (as determined by the length in basepairs of significant peaks) (Fig. 2a). The closest Arabidopsis homologs of H3K4me3 and H3K27me3-modified genes were divided into equally sized classes according to the length of the associated ChIP-seq peak. We identified significantly overrepresented biological processes associated with each class separately for H3K4me3 and H3K27me3, and then scored the representation of each GO term across the peak length classes. We chose four peak length classes to ensure a reasonable number of genes (870–3,275) were used for GO enrichment analysis, thus avoiding spurious enrichments. The GO terms were then clustered according to their pattern of representation across the peak length classes (2^4^–1 = 15 clusters possible), ranked by a relative peak length score for each pattern (shown schematically in Fig. 2a), and the number of terms for each cluster evaluated for inflation or deflation above expectation using a binomial test.

Interestingly, enriched biological processes differed markedly in number and composition across peak length clusters. For H3K4me3, the cluster with the highest peak length score (cluster 1) contained ~11-fold more terms than expected (*P* < 2.2 × 10^−16^), and was characterized by GO terms relating to xylogenesis (e.g. SCW biogenesis, cellulose biosynthesis, cell wall organization or biogenesis, phenylpropanoid biosynthesis, xylem and phloem pattern formation), as well as terms relating to early differentiation such as cell growth, tissue development, regulation of cell size and organ morphogenesis. Notable, these terms were absent from all other clusters with a lower peak length score (Fig. 2b; Supplementary Dataset 3). Programmed cell death was not enriched in any cluster, consistent with the sampling of early xylem development in this study. Similarly, for H3K27me3, the highest-scoring cluster was inflated for unique GO terms above expectation (~4-fold), where the first three clusters with higher peak length scores were clearly biased toward wood development processes such as cell wall modification, xylem development and lignin biosynthesis or metabolism, while phenylpropanoid biosynthesis was associated with mid-scoring cluster 7 which was also significantly enriched for GO term enrichment (*P* = 9.2 × 10^−10^) (Fig. 2b, Supplementary Dataset 4). Notably, gene length was not correlated with H3K4me3 (Pearson’s r = −0.053) or H3K27me3 (r = −0.07) peak length, ruling out the possibility that the biological enrichments may be biased by gene length. These results suggest that genes involved in secondary xylem development are likely to be H3K4- and/or H3K27-trimethylated at successive nucleosomes to a pronounced degree.

**Figure 3:**
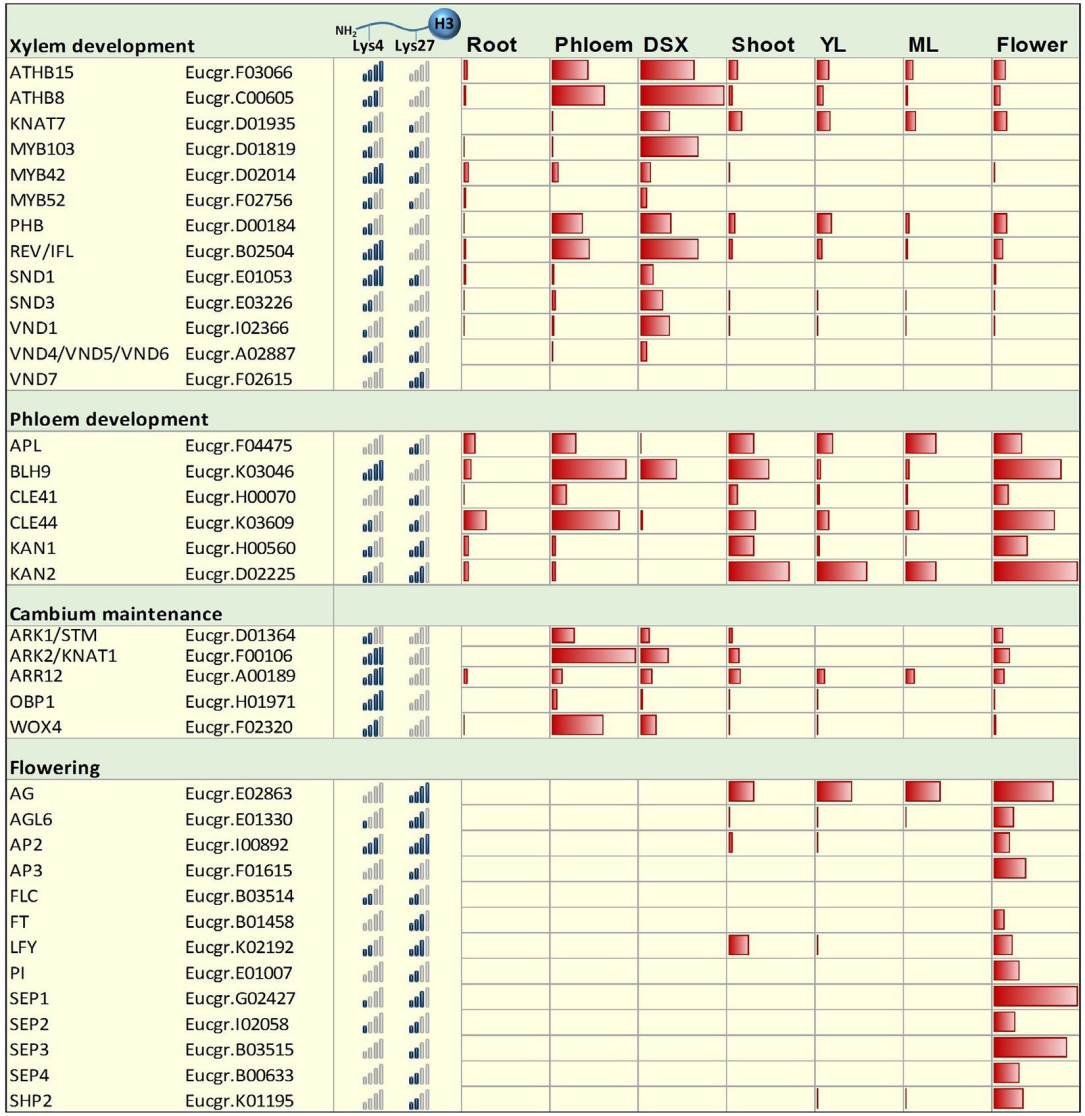
Relative enrichment of H3K4me3 and H3K27me3 for *E. grandis* homologs of transcription factors and signal peptides regulating vascular development and flowering in developing secondary xylem tissue. Blue bars, enrichment of H3K4 and H3K27 trimethylation (shown as N-terminal region of histone H3), represented as a signal of zero (no significant enrichment) to four bars (16-fold enrichment). Red bars, absolute RNA-seq expression levels (max FPKM value, 228) for roots, phloem, developing secondary xylem (DSX), shoots, young leaves (YL), mature leaves (ML) and flowers^41, 42^.

To further dissect the role of HMs and especially potentially bivalent epigenomic states in xylogenesis, we compared the relative signal enrichment of H3K4me3 and H3K27me3 marks for key transcription factors and signal peptides associated with vascular development and analysed their expression profiles across various tissues^41, 42^. Generally, transcription factors regulating xylem development (homologs of *ATHB8*, *ATHB15*, *REV*, *SND1*, *SND3*, *KNAT7*) as well as several cambial markers (*STM*, *KNAT1*, *ARR12*, *OBP1* and *WOX4*) had a much higher overall enrichment for H3K4me3 than H3K27me3, while for phloem markers (*APL*, *CLE41*, *CLE44*, *KANADI*) a stronger H3K27me3 signal was observed (Fig. 3a). Homologs of floral homeotic genes, which are generally strongly repressed^43–45^, are shown for comparison. Orthologs of xylem vessel developmental markers such as *VND1* and *VND4*/*VND5*/*VND6* also showed higher H3K27me3 signals, possibly owing to repression in fibre cells, especially the protoxylem marker *VND7* (Fig. 3a). Genes considered *bona fide* members of lignin biosynthesis^46^ were ~4.6-fold enriched for potentially bivalent genes (*P* = 1.93 × 10^−6^), while genes involved in SCW polysaccharide biosynthesis^20^ were not enriched.

### Tissue-specific expression of H3K4- and H3K27-trimethylated genes in developing secondary xylem

**Figure 4:**
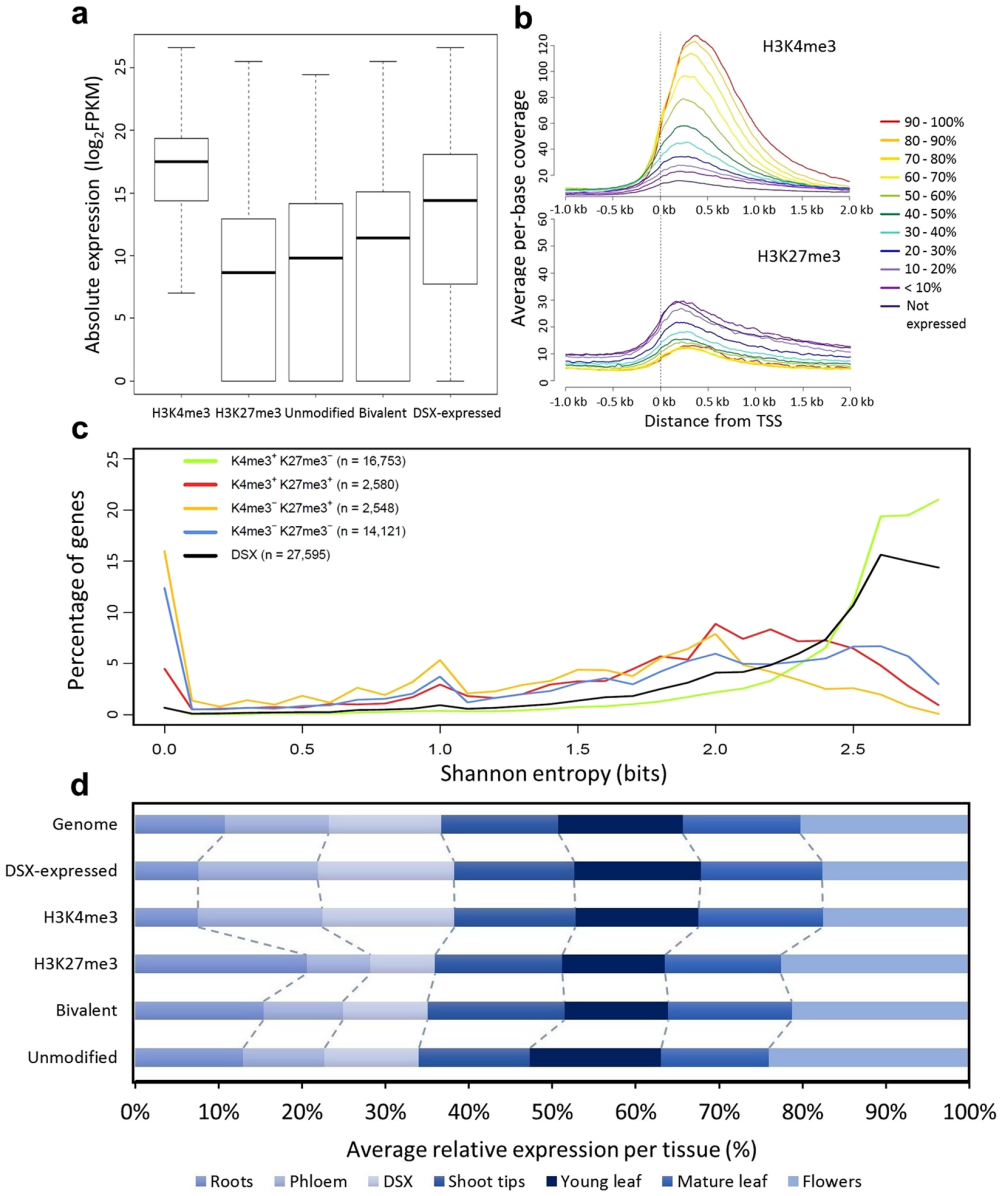
Association of H3K4me3 and H3K27me3 with gene expression levels and specificity. (**a**) Box plot of absolute expression values of genes from different epigenomic categories. The absolute expression profile for developing secondary xylem (far right) is shown for comparison. (**b**) Average per-base coverage of H3K4me3 (top) and H3K27me3 (bottom) ChIP-seq libraries around the transcription start site (TSS) of genes expressed at various levels in developing secondary xylem. (**c**) Tissue-specificity distribution of genes marked by different combinations of H3K4me3 and H3K27me3, as measured by the Shannon entropy index (calculated for genes with nonzero expression in at least one tissue). All pairwise combinations are statistically significant (Kolmogorov-Smirnov test, *P* < 2.2 × 10^−16^). (**d**) Average relative expression of genes in different epigenomic categories in roots, phloem, developing secondary xylem, shoot tips, young leaves, mature leaves and flowers. The relative expression in each tissue of all annotated genes in the genome and genes expressed in developing secondary xylem are shown for comparison. DSX, developing secondary xylem.

Based on transcriptome data for seven tissues and organs^41, 42^, for DSX tissue the H3K4-trimethylated genes in DSX had a high median expression level, whereas H3K27-trimethylated genes in DSX showed low expression levels in general (Fig. 4a). After genes were assigned to different expression level categories based on DSX RNA-seq data, the average coverage of H3K4me3 and H3K27me3 ChIP-seq libraries at 5′ transcribed regions increased and decreased, respectively, as expression levels increased (Fig. 4b). Unmodified (H3K4me3^−^ H3K27me3^−^) and potentially bivalent (H3K4me3^+^ H3K27me3^+^) genes were expressed at higher levels than those marked by H3K27me3 alone, but were still expressed below the median for DSX tissue (Fig. 4a). The tissue specificity of genes with different epigenomic combinations of H3K4me3 and H3K27me3 was assessed next, according to the Shannon entropy index^47^. Designating the entropy distribution of all genes expressed in DSX as a reference, genes exclusively trimethylated at H3K4 (H3K4me3^+^ H3K27me3^−^) had significantly higher entropy (Kolmogorov-Smirnov test, *P* < 2.2 × 10^−16^), which indicates broad expression, whereas unmodified (H3K4me3^−^ H3K27me3^−^) genes and any genes with H3K27 trimethylation (i.e. H3K4me3^+/−^ H3K27me3^+^) were associated with significantly lower entropy (especially for H3K4me3^−^ H3K27me3^+^ genes) indicating more tissue-specific expression (Fig. 4c, Supplementary Table S4, Supplementary Fig. S8).

The tissue-specific expression patterns of genes with different HM states was further explored (Fig. 4d). Compared to genes expressed in DSX, those marked by H3K4me3 in DSX showed a highly similar expression pattern across tissues (Pearson’s r > 0.99), while those marked by H3K27me3 were dissimilar (r < −0.26). The average relative expression of H3K27-trimethylated genes in vascular tissues (DSX, phloem) was significantly less than the value of genes expressed in DSX tissue (Pearson’s Chi-squared test, *P* < 0.0001), consistent with a repressive function (Fig. 4d). Interestingly, the most significant deviation from expectation was seen for the relative expression of H3K27-trimethylated genes in DSX, which was disproportionately augmented in roots compared to the remaining tissues (Pearson’s Chi-squared test, *P* < 0.0001; Supplementary Table S5). The significant enrichment of “response to hypoxia/oxygen levels” among H3K27me3 targets (previous section) corroborates the higher specificity for roots, suggesting targeted repression of genes involved in the development of this organ in DSX.

### Modelling steady-state transcript levels from histone modification data

The degree to which individual gene expression can be predicted by models of genome-wide chromatin modification data reveals insights into the biology of chromatin-level regulation, such as functional redundancies and the relative importance of different HMs in gene regulation. Despite diverse approaches to model transcript levels from ENCODE^48^, modENCODE^49^ and other HM data^17–19, 50, 51^, there are no reported attempts in plants to model transcript levels from HM data and thus the relative impact of known HMs on gene expression is yet to be quantified. Our ability to collect large amounts of DSX tissue from each tree allowed us to profile H3K4me3, H3K27me3 and transcript levels from the same DSX samples, providing an ideal opportunity to predict gene expression based on two antagonistic HMs. Thus, we generated strand-specific RNA-seq data from the two DSX replicates (Supplementary Table S6), followed by Yeo-Johnson transformation^52^ using an optimal λ parameter.

**Figure 5:**
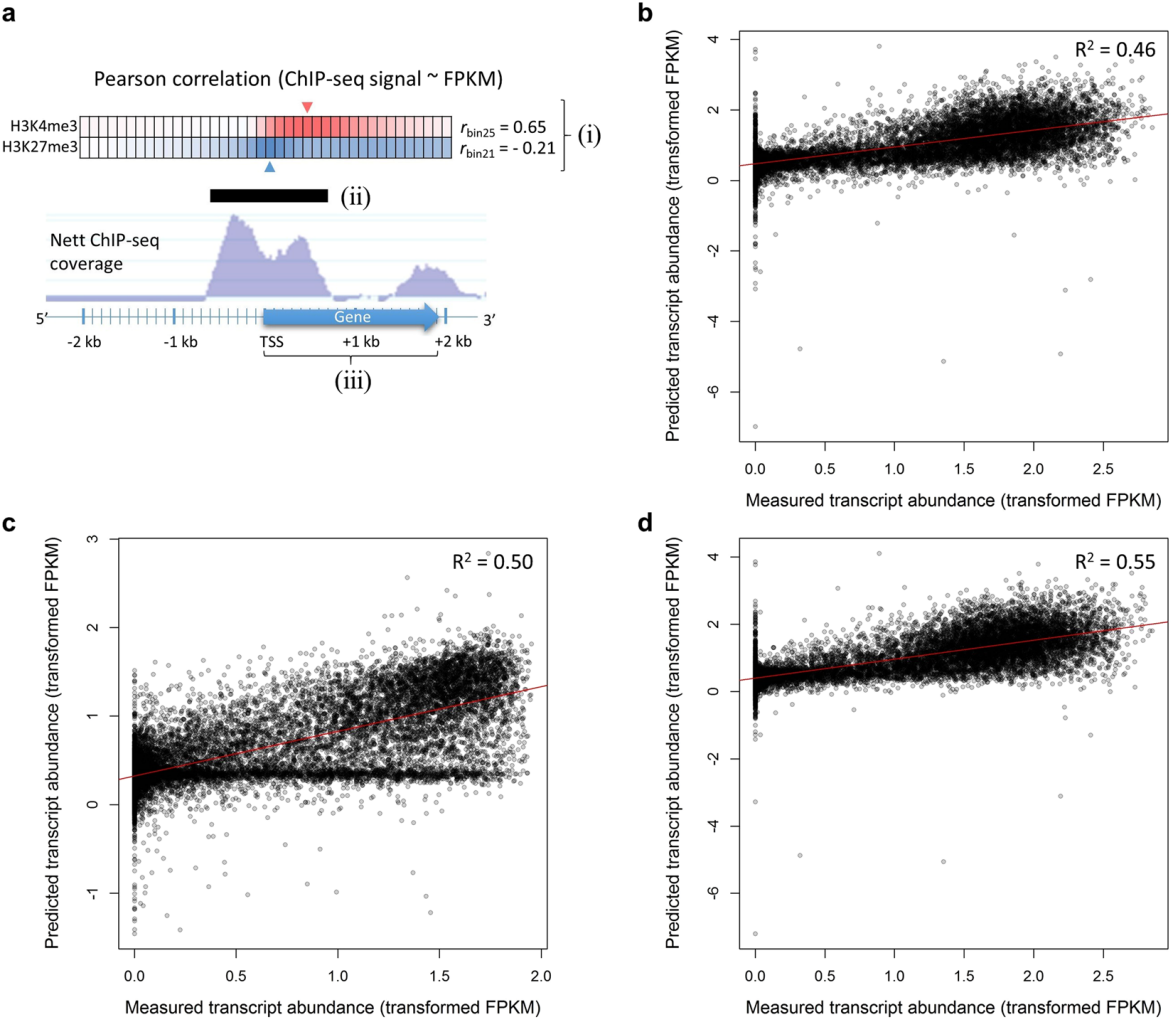
Prediction of steady-state transcript abundance in developing secondary xylem from histone modification ChIP-seq data in *E. grandis* and Arabidopsis. (**a**) Three ChIP-seq variables were considered for per-gene histone modification enrichment, namely the nett ChIP-seq coverage over one of forty 100 bp bins showing the strongest Pearson correlation with transcript abundance (i), the significant ChIP-seq peak length (bp) (ii), and the nett ChIP-seq coverage across the entire transcribed region (iii). (**b**) Prediction of transcript abundance using a multiple linear regression model trained on *E. grandis* H3K4me3(bin25) and H3K27me3(bin21) ChIP-seq data. (**c**) Prediction of transcript abundance using a multiple linear regression model trained in Arabidopsis H3K4me3(bin24) and H3K27me3(bin21) ChIP-seq data. (**d**), Prediction of transcript abundance using nine HM ChIP-seq datasets in Arabidopsis^35^.

The method used to quantify ChIP-seq signals has a significant effect on model performance^53^. We therefore considered three ways of quantifying per-gene ChIP-seq signal for the HM data (Fig. 5a): (i) ChIP-seq coverage in one of forty 100 bp bins, centred around the TSS, that correlates optimally with gene expression^54^ (see Methods), (ii) the length in basepairs of the H3K4me3 or H3K27me3 peak, for those peaks that overlap genes and (iii) ChIP-seq coverage across the entire transcribed region, normalized for gene length, here defined as total signal. For (i), H3K4me3 signal correlated positively with gene expression level at a maximum value (r = 0.65) at bin 25 (Supplementary Table S7, Supplementary Fig. S9a), congruent with its approximate binding position (Fig. 1a). The H3K27me3 modification signal was negatively correlated with gene expression levels across most of the bins, with the strongest association (r = −0.21) recorded at bin 21, immediately after the TSS (Supplementary Table S7, Supplementary Fig. S9b).

Using 60% of the annotated genes as a training set and 40% as a testing set, we used multiple linear regression analysis to predict the degree to which variation in transformed FPKM values could be uniquely explained by these signal parameters, based on eta-squared (ƞ^2^). From a total explained variance of R^2^ = 0.53, H3K4me3 signal at bin 25, H3K4me3 and H3K27me3 peak lengths and H3K27me3 signal at bin 21 each explained more unique variation than the total signal for each mark (Supplementary Table S8). Since peak length is highly influenced by peak-calling methods, we simplified the model by using only H3K4me3 signal at bin 25 and H3K27me3 bin 21 signal, both of which are easily calculated. In this two-variable model, H3K4me3 (ƞ^2^ ≈ 0.455; *P* < 2.2 × 10^−16^) and, to a much lesser degree, H3K27me3 (ƞ^2^ ≈ 0.036; *P* < 2.2 × 10^−16^), together predicted ~50% of steady-state transcript abundance (Fig. 5b).

To investigate whether the relative proportion of variance in transcript abundance explained by H3K4me3 and H3K27me3 is not specific to DSX tissue or *Eucalyptus*, we repeated our modelling approach with published ChIP-seq data of these HMs in aerial tissues of Arabidopsis seedlings^35^, using RNA-seq data for similar tissues from plants grown under near-identical conditions^55^. We found that the expression data correlated optimally at a similar bin position and to a similar degree to that observed in our study (Supplementary Table S7), reinforcing the known evolutionary conservation of epigenomic biology. Compared to *E. grandis*, a similar degree of unique variation in transcript abundance was explained by H3K4me3 (ƞ^2^ ≈ 0.464; *P* < 2.2 × 10^−16^) and H3K27me3 (ƞ^2^ ≈ 0.017; *P* < 2.2 × 10^−16^), together predicting ~46% of measured transcript abundance in the testing set (Fig. 5c). The slightly lower correlation in Arabidopsis may be due to the fact that expression and HM data were not derived from the same experiment or tissue types as in our study.

An important remaining question is whether the integration of additional HMs can significantly increase transcript abundance predictions. To address this, and to quantify the unique predictive contributions by different HMs, we considered seven additional HMs generated from the same Arabidopsis study^35^. Bin-wise expression correlations peaked shortly after the TSS for all HMs aside from H3K36me2 (Supplementary Table S9). Despite high correlations with transcript abundance for many of the marks, multiple linear regression analysis revealed that the seven additional HMs boosted the transcript abundance prediction accuracy by only ~8.7%, totalling ~55% (Fig. 5d). The unique variation explained by H3K4me3 dropped to ƞ^2^ ≈ 0.096, followed by H3K9ac (ƞ^2^ ≈ 0.036) as well as H3K18ac (ƞ^2^ ≈ 0.025) and H3K27me1 (ƞ^2^ ≈ 0.023) despite a weak correlation with transcriptome data (Supplementary Tables S9 and S10). Redundant information between some marks, for example between H3K4me2, H3K36me2 and H3K36me3^35^, may explain the higher relative informativeness of HMs that have a weaker association with gene expression (Supplementary Table S9). The finding of a high degree of interaction between H3K18ac and H3K27me3^35^ may explain the low unique variation explained by H3K27me3 when considering these marks simultaneously in a model. Together, these results show that the predictive value of H3K4me3 and H3K27me3 in modelling gene expression is highly conserved across the evaluated lineages and tissue types, that H3K4me3 captures the most epigenomic information on gene expression consistent with its representation of transcription initiation, and that predictive models are improved most, although modestly, by the inclusion of apparently minor but informative HMs.

## Discussion

In this study, we aimed to understand the functions of major activating and repressing HMs in the chromatin architecture of early-differentiating secondary xylem and quantify their value as predictive markers of gene transcript levels in plants. We found that, while H3K4me3 and H3K27me3 are deposited at genes enriched for generalized and specific biological processes, as well as non-tissue-specific and tissue-specific expression respectively, genes occupied by the broadest signals show a clear bias for xylogenesis-associated terms for both marks. H3K4me3 signal is far more informative than H3K27me3 in reflecting absolute expression levels across the genome, while it seems that H3K27me3 is deposited at transcriptionally repressed, tissue-specific and especially tandemly duplicated subsets of genes. The latter may indicate a role for H3K27me3 in the pseudogenization of tandem duplicates and remains to be further investigated. That around 750 H3K27-trimethylated genes in DSX are homologs of previously unknown H3K27me3 targets in plants is hardly surprising given that woody vascular tissue is unique and somewhat more homogeneous than complex organs used in most epigenetic studies in herbaceous models such as Arabidopsis and rice. In a study of five maize tissues, approximately 40% of H3K27me3-occupied genes were unique to each tissue^56^. Thus, we anticipate that considerably more H3K27-trimethylated genes, currently encompassing around half of those annotated in Arabidopsis, will be discovered as new tissues and cell types are explored in ChIP studies.

We were intrigued to find many xylogenesis-associated transcription factors to be in a potentially bivalent state, associated with low-level, tissue-specific expression (Fig. 4a). H3K4me3 and H3K27me3 modifications can co-occur at the same nucleosome^8^, unlike H3K27me3 and H3K36me3^57^. However, Luo *et al*.^35^ found using reciprocal H3K4me3 and H3K27me3 re-ChIP assays that only around half of the bivalent gene candidates tested were truly bivalent chromatin fibres, where even for those loci it is likely to be the case for only a proportion of cells sampled. Thus, the potentially bivalent regions may reflect mixed signals of activating HMs in some DSX cell types (e.g. fibres) and repressive HMs in others (e.g. vessels) within the same tissue. In this scenario, one would expect that false bivalent HM signals brought about by a mixture of cell types will result in an H3K27me3 signal lower than the median due to a dilution effect; however, in our study the median H3K27me3 fold-change for potentially bivalent genes (≈7.9) was similar to that of all H3K27-trimethylated genes (≈7.9), therefore not supporting this hypothesis.

Bivalency may arise as a temporary transition state from one chromatin mark to another. This could include H3K4me3-to-H3K27me3 transition, observed in seed developmental genes during germination^58^. Alternatively, bivalent regions may result from a transition from repressed (H3K27me3) to activated (H3K4me3) epigenetic states at TF genes that promote cambium differentiation into xylem. This is known to occur during leaf-to-callus dedifferentiation, where pluripotency is mediated by H3K27me3 silencing of leaf differentiation genes and de-repression of auxin pathway genes^34^, and in animals, where developmentally important TFs required for post-embryonic development are kept repressed by H3K27me3 but “poised” for rapid activation via H3K4me3^59, 60^. We noted that TFs associated with xylem and phloem development, but not vascular cambium, were frequently found within potentially bivalent chromatin domains (Fig. 3). Since our collected tissue included cambium, xylem mother cells and the earliest stages of fibre and vessel development, we would expect this pattern if TFs promoting differentiation were silenced by H3K27me3 in the vascular cambium and gradually activated by H3K4me3 during early differentiation. Furthermore, we also found a distinct enrichment among the 38 expressed *bona fide* lignification genes but not SCW polysaccharide genes in *E. grandis* ^20, 46^, substantiated by an enrichment for H3K27me3 among lignin and phenylpropanoid biosynthesis only among genes in two H3K27me3 peak-length clusters (Fig. 2b). Since most of the lignification occurs *post mortem* after SCW polysaccharide deposition^61^, and hence at a later developmental stage than the tissue that was sampled, we can expect a distinct enrichment of H3K27me3 among these genes as they await transition from epigenetic repression to activation.

Benayoun *et al*.^62^ showed that cell identity markers that perform key roles in specifying cell fate tend to have broad H3K4me3 deposition in humans. A similar trend among photosynthesis-related genes during leaf development in plants suggests that key tissue-specific pathways may also be targeted for extensive H3K4-trimethylation^40^. Consistent with this, we observed evidence for a link between xylogenesis-associated terms and genes with the broadest peaks for both H3K4me3 and H3K27me3 (Fig. 2b). Linked to the biological significance of HM span, H3K4me3 and H3K27me3 peak lengths contributed significant unique information to gene expression predictions (Supplementary Table S8). Peak length may be informative in cases where the ChIP-seq signal has reached saturation (since there are only two residues per nucleosome available for each HM), and the mark has spread to adjacent nucleosomes. Thus, the establishment of H3K4me3 and H3K27me3 at successive nucleosomes may be linked to distinct biological processes.

Despite several studies using HM and transcription factor binding data to model gene expression in animals and yeast, our understanding of the transcriptional effects of HMs in plants is poor. Studies in animal models have shown that the predictive value of HM data on gene expression is largely redundant with transcription factor binding site data^51, 54, 63^, demonstrating the utility of HMs as indicators of transcriptional states. While the causal relationship between HMs and transcription remains a “hen-and-egg” problem in epigenetics^64, 65^, predictive modelling can reveal redundancies, dependencies and antagonisms between HMs. Our study quantified the impact of HM status on gene expression in both *E. grandis* and Arabidopsis, not only for H3K4me3 and H3K27me3 as active and repressed chromatin representatives, but also in the context of seven additional HMs. We confirmed that 100 bp region-specific H3K4me3 and H3K27me3 signals, both near the TSS, are more informative than average signals across transcribed regions for plants, as shown in non-plant organisms^18, 66, 67^. The total expression variation explained by region-specific H3K4me3 and H3K27me3 in *E. grandis* (~50%) is outstanding for such a small number of HMs: the best-performing two-modification model out of 820 HM combinations in CD4^+^ T-cells produced R^2^ ≈ 0.55^17^. Our parallel analysis in Arabidopsis showed a similar contribution by H3K4me3 and H3K27me3, despite the stark difference in the tissues and species compared. However, it is likely that the relative informativeness of other HMs will change across tissues and environmental conditions. The much smaller predictive value of H3K27me3 relative to H3K4me3 in both organisms is not surprising considering the smaller number of genes significantly enriched for H3K27me3 (15.9% of genes in *E. grandis* versus 53.9% of genes for H3K4me3), but redundant information between these antagonistic marks could also lower the unique variation explained by each. It is because of this redundancy that vastly diminishing returns are obtained when considering additional HMs in predictive models, as demonstrated by the modest increase in model performance when we included data for seven additional HMs in Arabidopsis. Nonetheless, our model accuracies exceeded several non-plant studies such as the support vector approach by Budden *et al*.^51^ using six HMs and DNase-seq data in human GM12878 cells which yielded an R^2^ of 0.45 – smaller than our two-HM Arabidopsis models – or the Multivariate Adaptive Regressive Splines model of Xu *et al*.^68^ who obtained R^2^ ≈ 0.52 from twenty-one HMs in CD4^+^ T-cells including their interactions, versus R^2^ ≈ 0.55 from our nine Arabidopsis HMs.

The roles of HMs in the epigenetic regulation of gene expression in plants are diverse and far from understood. H3K4me3 and H3K27me3 appear to be associated with distinct biological processes during secondary xylogenesis in the *E. grandis* model, as revealed by their presence and genomic span along target genes. The predictive value of H3K4me3 and H3K27me3 in gene transcription in *E. grandis* DSX is conserved in Arabidopsis, where transcript abundance can be predicted using nine HMs with an accuracy meeting or exceeding most modelling approaches in non-plant organisms. The significance and regulatory functions of potentially bivalent chromatin domains enriched for particular biological pathways, among them developmentally important transcription factors regulating SCW formation and lignin biosynthesis, as well as the true bivalent status of these loci, remain to be further elucidated. Fine dissection of epigenetic profiles in vascular tissues, such as along xylogenesis developmental gradients and ultimately at single cell level, are challenging future prospects towards addressing this question.

## Materials and Methods

### Plant materials

DSX tissue was collected as described previously^21^ from two eight-year-old ramets of *E. grandis* clone TAG0014 (Mondi Tree Improvement Research, KwaMbonambi, South Africa), grown in a trial in KwaMbonambi, South Africa and sampled in April 2014. The tissue was fixed at room temperature in fixation buffer containing 1% formaldehyde for 15 min in the field, quenched and rinsed twice (Kaufmann *et al*., 2010) prior to flash-freezing in liquid nitrogen. Some of the (unfixed) tissue was flash-frozen for RNA extraction.

### ChIP-seq sample preparation, sequencing and data analysis

The DSX tissue was finely ground in liquid nitrogen and chromatin was extracted as described in Li *et al*.^69^. ChIP was performed with ~2 μg anti-H3K4me3 (Merck Millipore #07–473), anti-H3K27me3 (Merck Millipore, ABE44) or anti-RNA Pol II (Merck Millipore, #17–672) antibodies using the protocol described in Adli and Bernstein^70^, without ChIP DNA amplification. Illumina ChIP-seq libraries of immuno-enriched and input samples were prepared and sequenced (SE50 reads; 200 bp library insert) by the Vincent L. Coates Genomics Sequencing Laboratory, University of Berkeley, CA. Read quality assessment and mapping to the *E. grandis* genome v.1.1^20^ was performed as described in Hussey *et al*.^21^. Strand cross-correlation analysis was performed as described in Landt *et al*.^22^. Significantly enriched regions were identified using the ENCODE Irreproducible Discovery Rate (conservative) method with a threshold of 0.01^66^ on peaks identified using MACS v.2^71^. Peaks overlapping with those identified using a nonspecific antibody^21^ were discarded. Genes were regarded as modified for each HM if the annotated transcribed regions overlapped a significant ChIP-seq peak to any degree.

### Dot blot analysis

Peptides representing various lysine methylation states of H3K4me3 (MARTKQTAR) and H3K27me3 (VARKSAPA) were synthesized (GenScript Ltd, Hong Kong) and blotted onto nitrocellulose. The membrane was blocked and probed with anti-H3K4me1 (ABE1353), anti-H3K4me2 (ABE250), anti-H3K4me3 (07–473) or anti-H3K27me3 (ABE44; Merck Millipore), then anti-rabbit HRP-conjugated secondary antibody and exposed to film using SuperSignal West Pico Chemiluminescent substrate (Thermo Scientific, Rockford, IL).

### RNA sequencing

Total RNA was extracted from DSX tissue, stranded Illumina RNA-seq libraries were prepared, and PE50 reads generated (Illumina HiSeq2500) by Beijing Genome Institute, Hong Kong. Reads were filtered (phred score >20 for ≥50% of the read, ≤10% “N” bases), assessed for quality using FastQC (http://www.bioinformatics.babraham.ac.uk/), mapped to the *E. grandis* genome using Tophat2^72^ (sensitive mode; intron length 50 bp to 30 kb; number of threads, 4), and FPKM values calculated for the longest predicted transcript with Cufflinks^73^. Arabidopsis RNA-seq data was obtained from Liu *et al*.^55^ as raw reads (SE101), which were trimmed to 91 nt to correct for base composition bias, filtered for quality, aligned to the Arabidopsis TAIR8^74^ genome and FPKM values similarly calculated as the average of three biological replicates.

### Bioinformatics and statistical analyses

ChIP-seq library coverage at the TSS and Shannon entropy indices were calculated as described previously^21^ using RNA-seq data from roots, DSX, phloem, shoot tips, young leaves, mature leaves and whole flowers for three five-year-old *E. grandis* trees^41, 42^. For enrichment and depletion analyses (transposable elements), a two-tailed hypergeometric test was used where the expected value for each category was calculated as the proportion of all genes marked by H3K4me3 or H3K27me3. Gene Ontology enrichment analysis was conducted with the Cytoscape BiNGO plugin^75, 76^, using as reference the Arabidopsis homologs of all *E. grandis* genes. For biological process enrichment analysis of genes occupied by HM peaks of differing lengths, the closest Arabidopsis homologs of H3K4me3 and H3K27me3-modified genes were divided into equally sized classes according to the length of the associated ChIP-seq peak. Significantly overrepresented biological processes within each class were scored for their representation (present, 1, or absent, 0) across the peak length classes. From the patterns of representation across the classes, 16 patterns (clusters) are possible, minus the class where no representation is observed in any cluster, thus totalling 15 clusters. A weighted peak length score was assigned to each cluster by multiplying each term’s presence or absence score (1 or 0) with 0.1, 0.2, 0.3 or 0.4 for peak length class 1 through 4, respectively, and dividing the sum by the total number of matches for each cluster. A binomial test of term enrichment for each cluster was performed in R using the formula, binom. test (x, n, p = (1/15), alternative = “two.sided”), where x is the number of terms matching the cluster, n is the total number of terms across all clusters, and the p is the theoretical probability of a match to a cluster. For Chi-squared analysis of tissue specificity of different gene groups (Fig. 4d), average relative expression was discretized by counting the number of genes in a given group and tissue with maximum expression observed in that tissue. A global Chi-squared test was performed, reporting post-hoc adjusted standardized residuals for each tissue. All statistical tests in this study, where applicable, are two-sided.

### Multiple linear regression modelling

Nett ChIP-seq coverage (i.e. average per-base coverage of the treatment profile minus the input control profile) for HMs over different genomic regions were calculated from bedGraph data files generated by MACS v.2^71^. Arabidopsis ChIP-seq data (BED format), obtained from Luo *et al*.^35^, were converted to bedGraph format and similarly processed using the TAIR8 annotation. To obtain region-specific HM enrichment data, each gene was divided into forty 100-bp bins centred at the TSS (±2 kb) as indicated in Fig. 5a, and nett ChIP-seq coverage for each bin was calculated using BEDTools^77^. ChIP-seq coverage in the bin showing the highest correlation between nett ChIP-seq coverage and transcript levels (Supplementary Table S7) was considered the region-specific HM enrichment value. Total signal was defined as the nett ChIP-seq coverage across the entire annotated transcribed region, normalized by gene length to avoid gene length bias. Expression data as Fragments Per Kilobase of exon per Million mapped reads (FPKM) values^78^ of the processed Arabidopsis and *E. grandis* RNA-seq data were transformed using the Yeo-Johnson method^52^, in the “boxCox” function of the “car” package^79^, with an empirically determined λ exponent of −0.516 for *E. grandis* and −0.336 for Arabidopsis. Multiple Linear Regression models were trained on approx. 60% of annotated genes and validated on the remaining 40% using the “lm” function in the “stats” R package^80^. The contribution to explained variance was measured using the “etasq” function from the “heplots” package (http://CRAN.R-project.org/package=heplots).

## Electronic supplementary material

Supplementary information

Supplementary dataset 1

Supplementary dataset 2

Supplementary dataset 3

Supplementary dataset 4
