# Supplementary material for: Integrated analysis and transcript abundance modelling of H3K4me3 and H3K27me3 in developing secondary xylem
Source: Sci Rep. 2017 Jun 13;7:3370. doi: 10.1038/s41598-017-03665-1 (PMC5469831; doi:10.1038/s41598-017-03665-1)
Supplement: Supplementary file 1 — Supplementary information [file 41598_2017_3665_MOESM1_ESM.pdf]

Supplementary Information:

Integrated analysis and transcript abundance modelling of H3K4me3 and H3K27me3 in developing secondary xylem

Steven G. Hussey[1*], Mattheus T. Loots[2], Karen van der Merwe[3], Eshchar Mizrachi[1] and Alexander A. Myburg[1]

[1]Department of Genetics, Forestry and Agricultural Biotechnology Institute (FABI), [2]Department of Statistics, [3]Centre for Bioinformatics and Computational Biology, Genomics Research Institute (GRI), University of Pretoria, Private Bag X20, Pretoria 0028, South Africa

*Corresponding author email: steven.hussey@up.ac.za

Supplementary figures

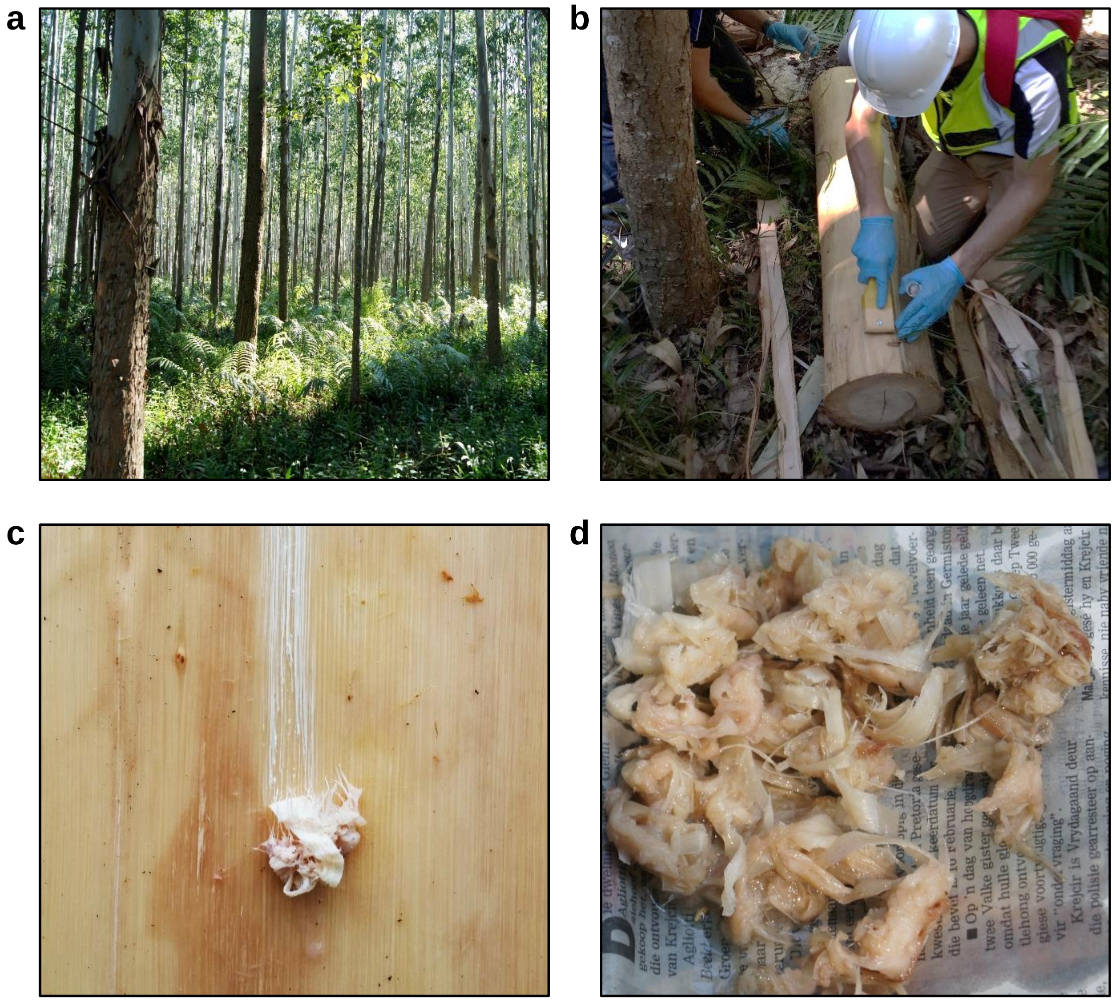

Figure S1. Tissue collection. Seven-year-old field grown *Eucalyptus grandis* trees **(a)** were felled. The bark was removed piecemeal **(b)**. The developing secondary xylem layer **(c)** was sampled with a sharp scraper and immediately fixed in cold fixing buffer containing 1% formaldehyde. After quenching of the fixative, the tissue was briefly dried on newspaper **(d)** before flash-freezing in liquid nitrogen. Images were taken by Mr. Marius Laubscher (a, b), Mr. Rikkert Myburg (c) and Dr. Steven Hussey (d). All images © Forest Molecular Genetics Programme, University of Pretoria.

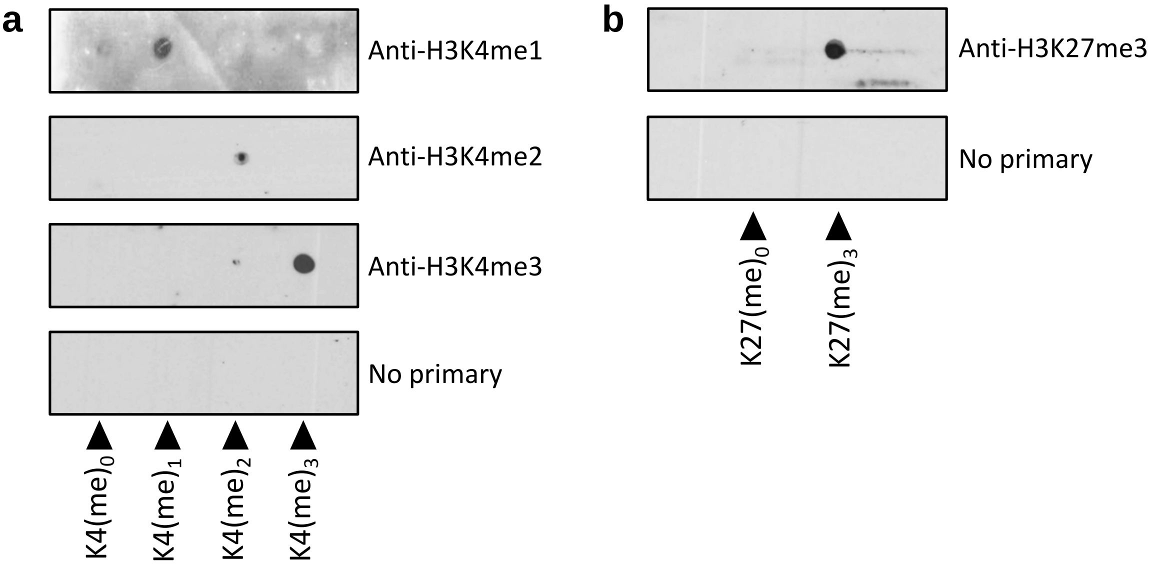

Figure S2. Dot blot analysis of anti-H3K4me3 and anti-H3K27me3 antibodies. (a) Immunoblots of synthetic peptides representing non-methylated and mono-, di- or tri-methylated H3 lysine-4 variants proved with primary antibodies specific for each methylation pattern. **(b)** Immunoblot of non-methylated and tri-methylated H3 lysine-27 synthetic peptide probed with anti-H3K27me3 antibody.

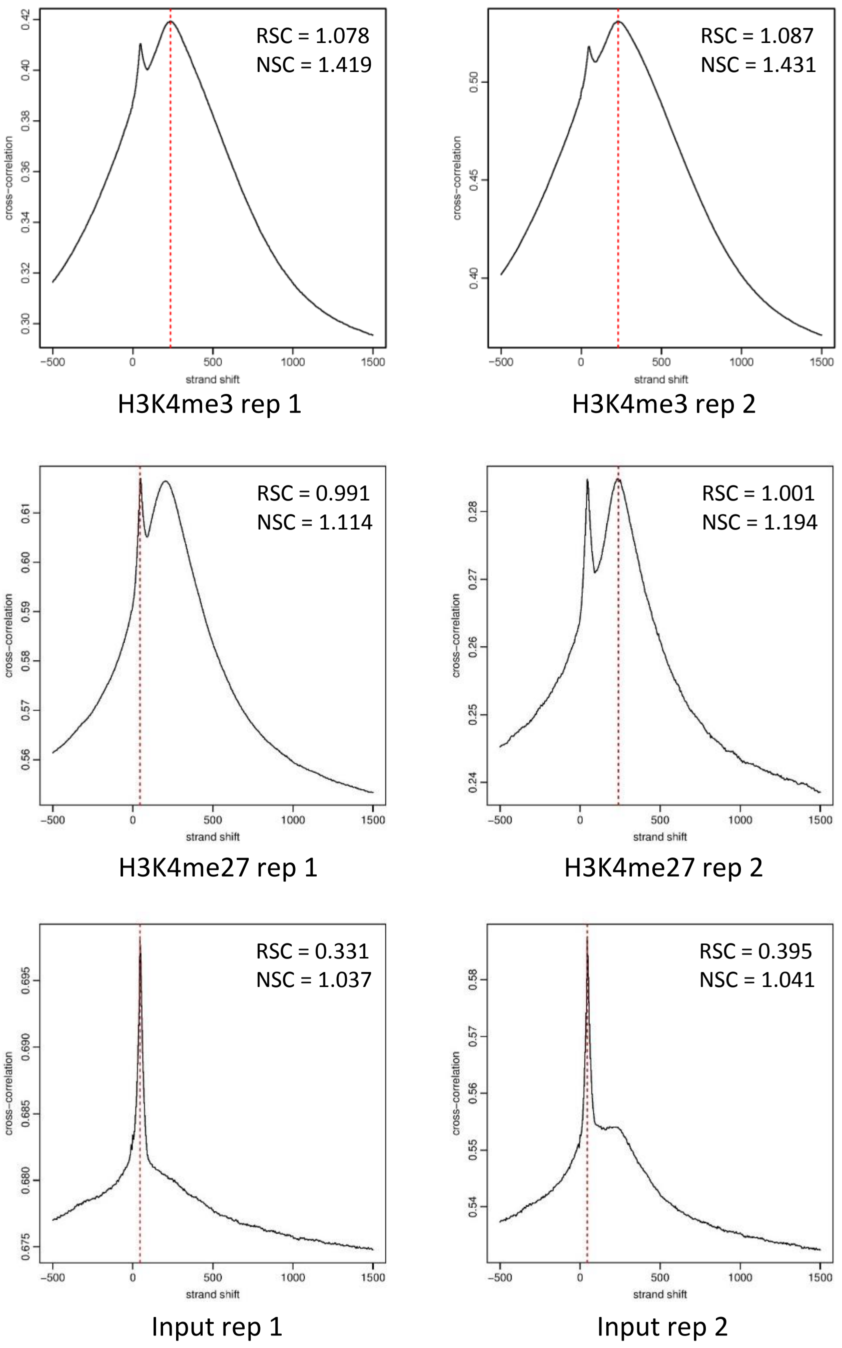

Figure S3. Cross-correlation analysis of H3K4me3 and H3K27me3 ChIP-seq libraries relative to input control. RSC and NSC ratios, defined in a previous work[1], should exceed 0.8 and 1.05 respectively for ENCODE immuno-enriched libraries but not input controls[2]. The cross-correlation profiles for all H3K4me3 and H3K27me3 replicates exceed these recommendations.

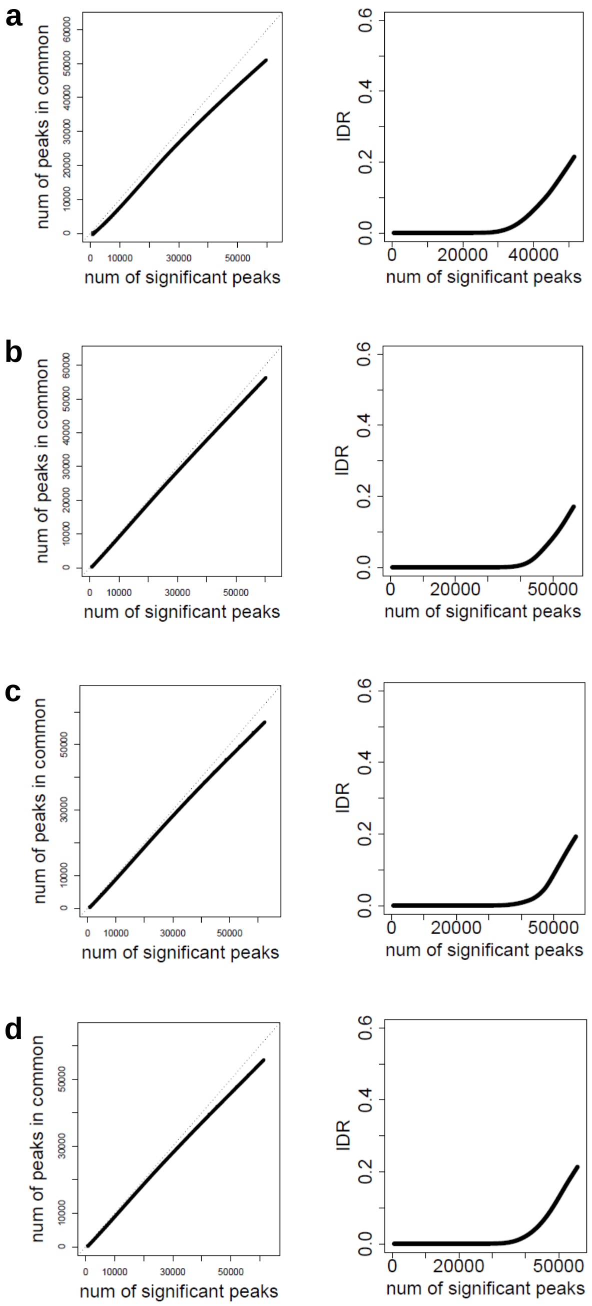

Figure S4. Biological reproducibility of H3K4me3 ChIP-seq peaks. The number of overlapping peaks as a function of increasing number of peaks identified (left) and the corresponding irreproducible discovery rate (IDR) for increasing numbers of identified peaks (right) is shown for biological replicates **(a)**, pseudoreplicates of bulked ChIP-seq data **(b)**, self-pseudoreplicates of biological replicate 1 **(c)** and self-pseudoreplicates of biological replicate 2 **(d)**.

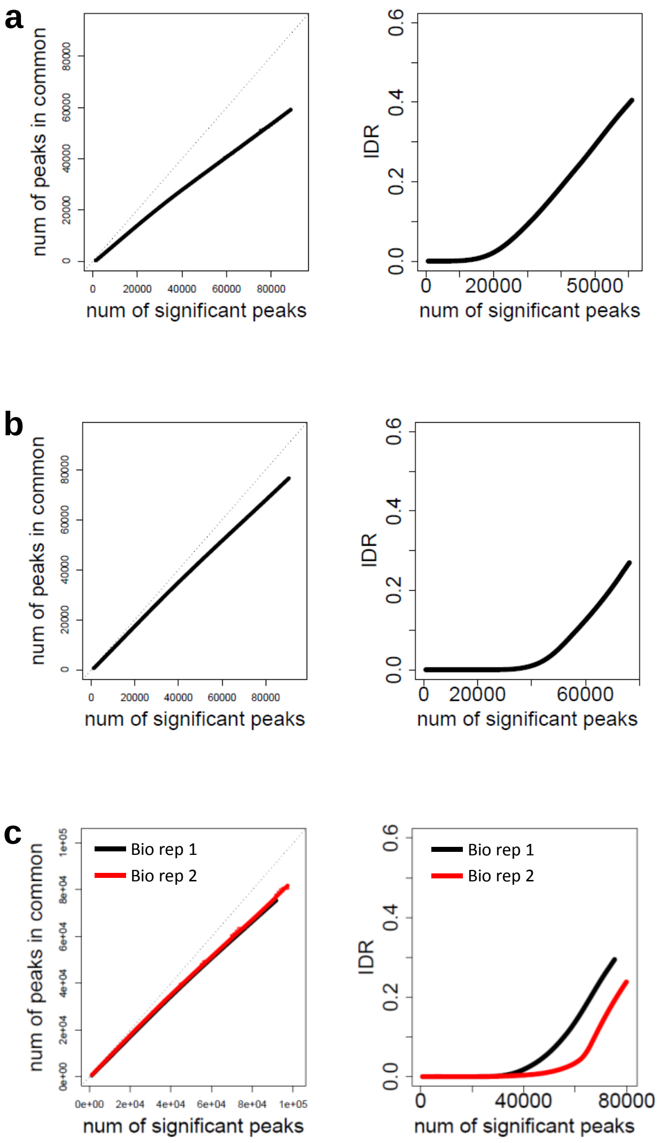

Figure S5. Biological reproducibility of H3K27me3 ChIP-seq peaks. The number of overlapping peaks as a function of increasing number of peaks identified (left) and the corresponding irreproducible discovery rate (IDR) for increasing numbers of identified peaks (right) is shown for biological replicates **(a)**, pseudoreplicates of bulked ChIP-seq data **(b)** and self-pseudoreplicates of biological replicates **(c)**.

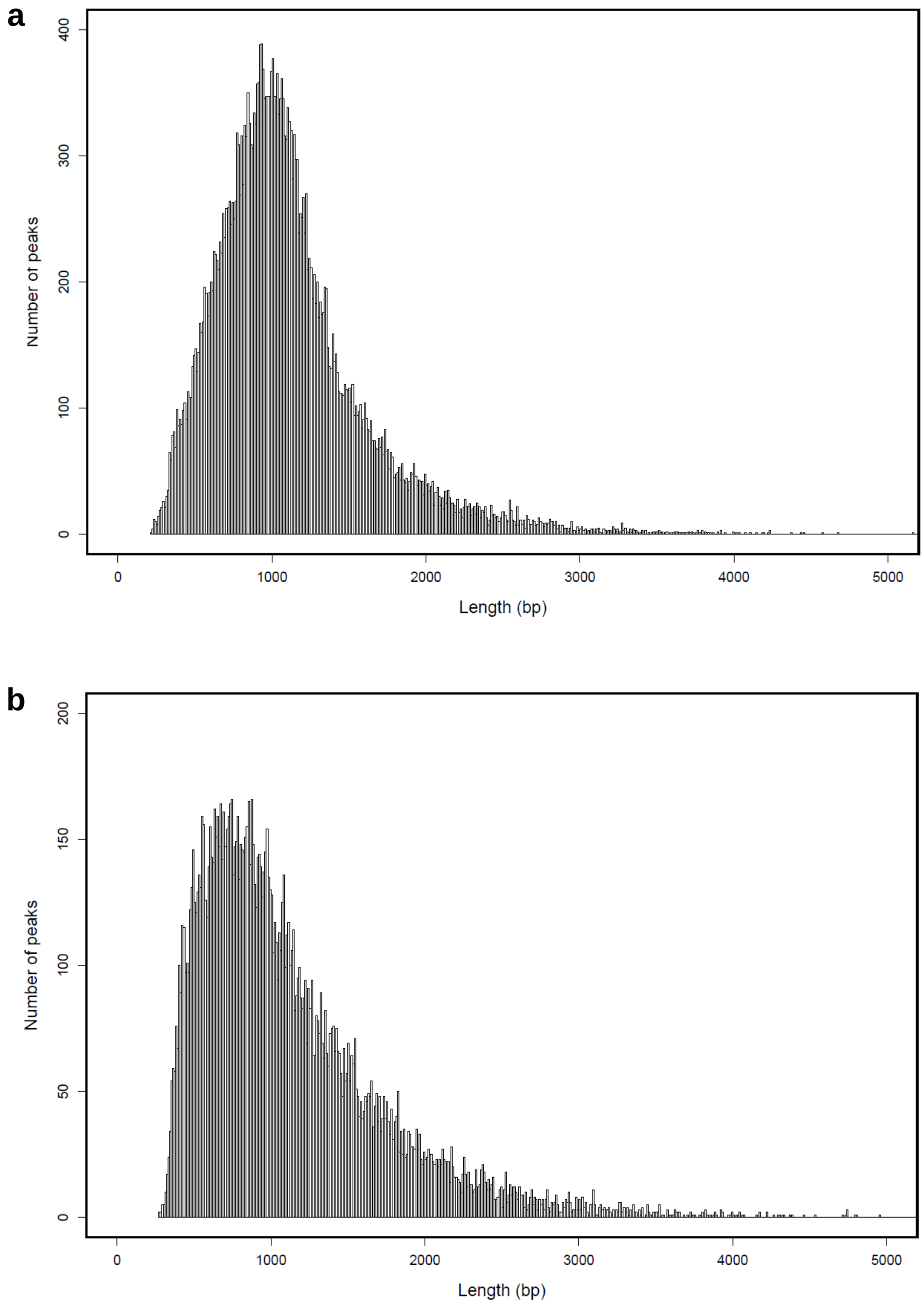

Figure S6. ChIP-seq peak length distributions. (a) Significant H3K4me3 peaks. **(b)** Significant H3K27me3 peaks.

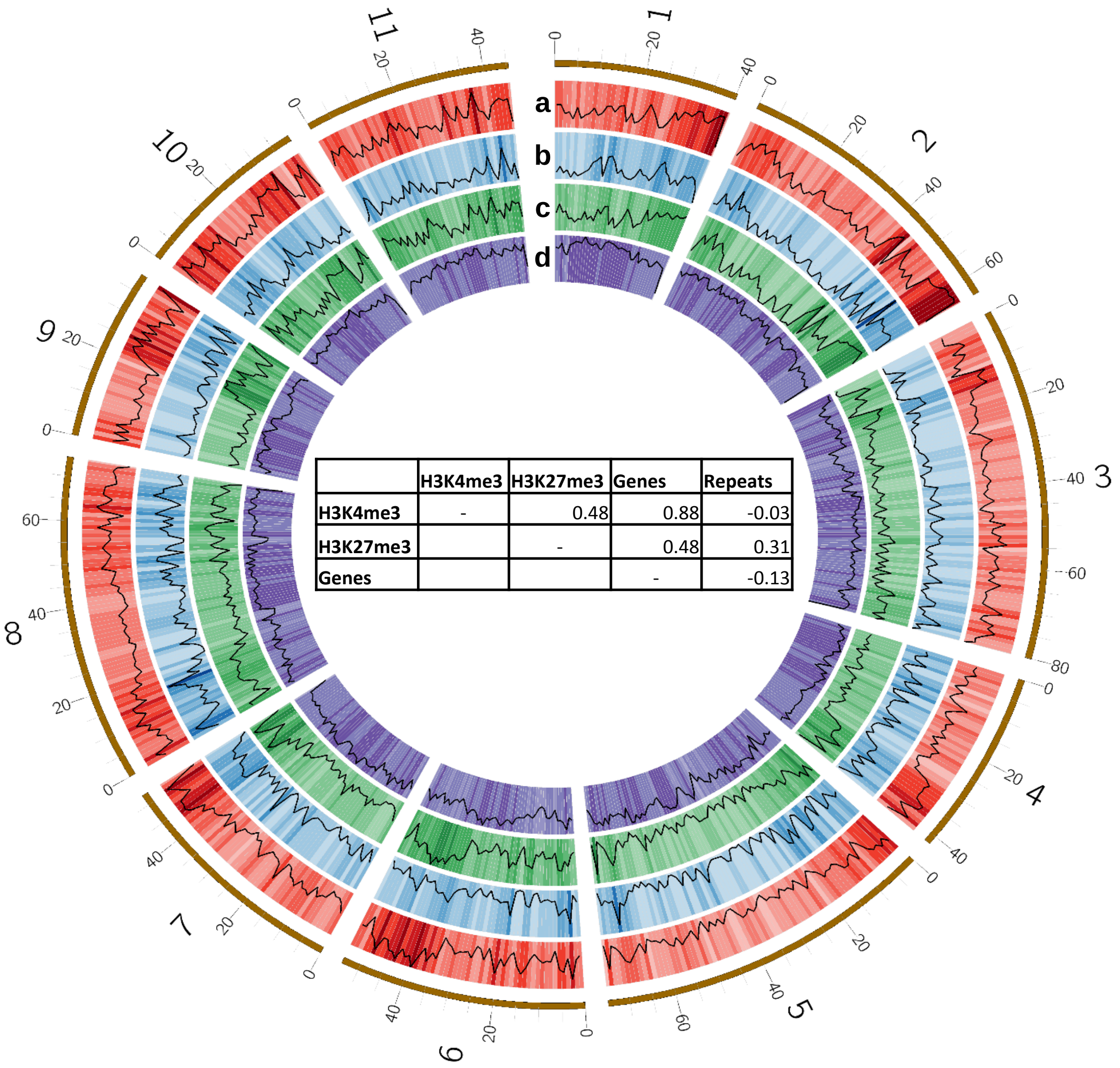

	H3K4me3	H3K27me3	Genes	Repeats
H3K4me3	-	0.48	0.88	-0.03
H3K27me3		-	0.48	0.31
Genes			-	-0.13

Figure S7. Circos graph of feature density per megabase. (a) H3K4me3 peaks; **(b)** H3K27me3 peaks; **(c)** genes and **(d)** masked genomic repeats**.** The table in the centre indicates the genome-wide Pearson correlation between each feature.

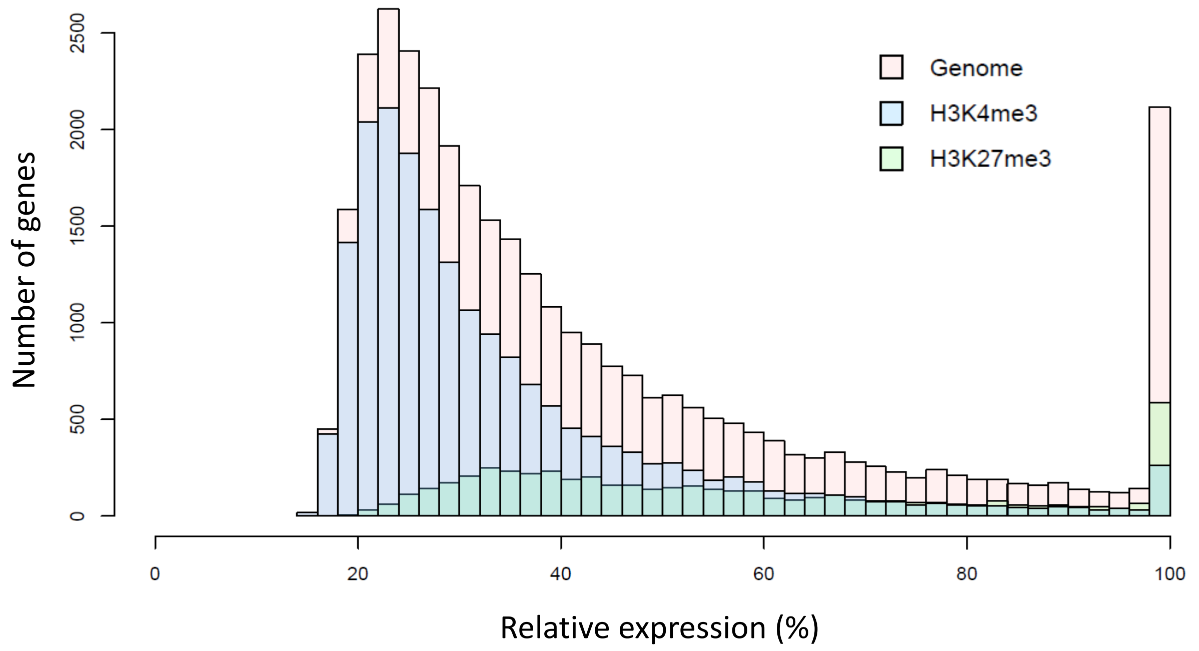

Figure S8. Histogram of maximum relative expression observed across seven tissues and organs for genes associated with H3K4me3 and H3K27me3.

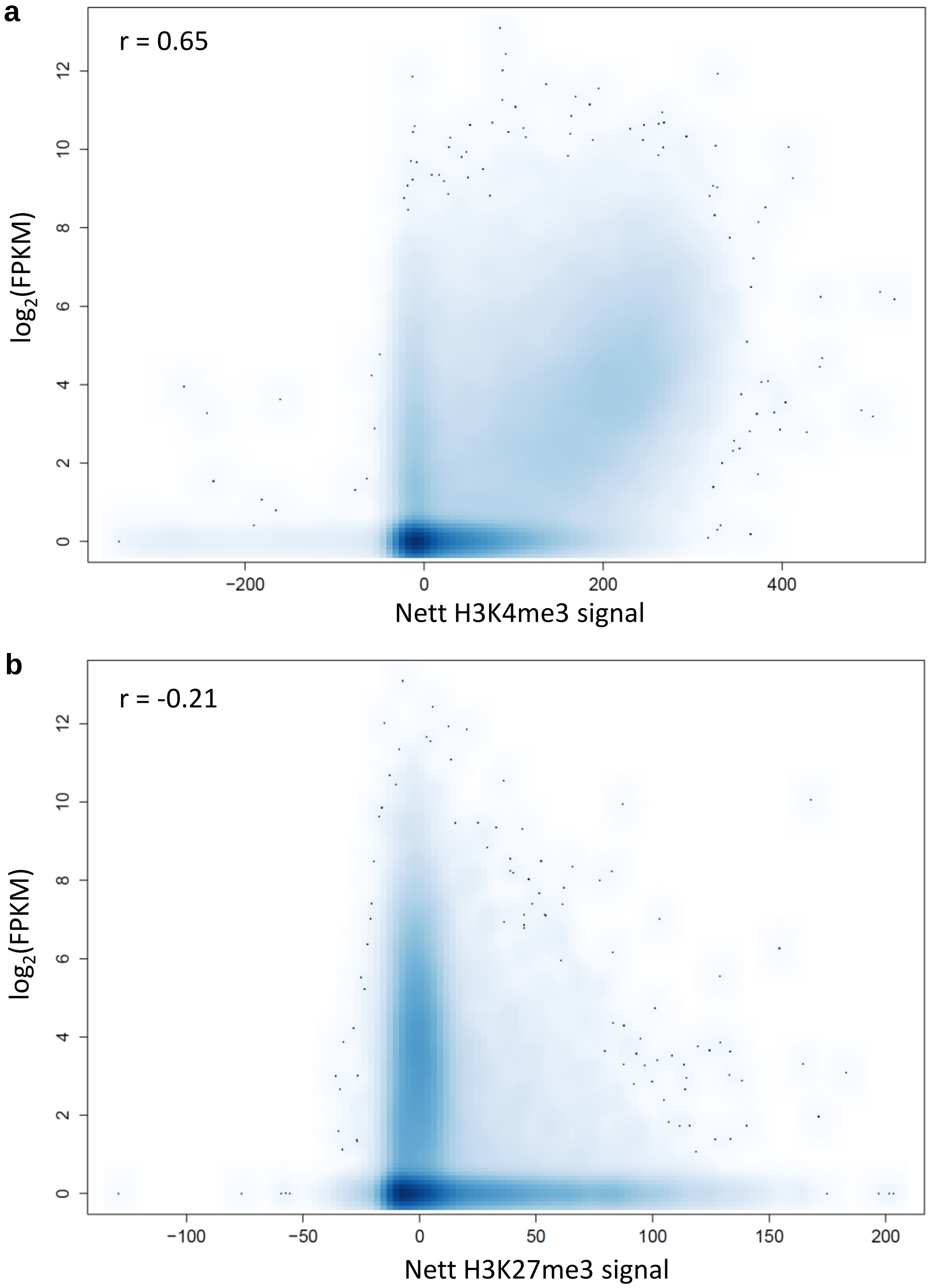

Figure S9. SmoothScatter plot of log-transformed FPKM values and histone modification signal. (a) H3K4me3 at bin 25. **(b)** H3K27me3 at bin 21.

Supplementary tables

Table S1. ChIP-seq library reads and mapping efficiencies.

Library	Filtered sequences (SE 50)	Mapped reads	Mapping rate	% aligning to plastid genome
H3K4me3 Rep 1	49 806 221	35 843 621	72.0%	0.2%
H3K4me3 Rep 2	42 632 238	34 332 958	80.5%	0.1%
H3K27me3 Rep 1	79 154 175	53 708 383	67.9%	0.5%
H3K27me3 Rep 2	42 891 103	20 489 147	47.8%	0.2%
Input Rep 1	67 882 119	55 598 391	81.9%	7.0%
Input Rep 2	44 074 115	34 670 447	78.7%	4.8%

Table S2. Transposable elements associated with H3K4me3 and H3K27me3 in developing secondary xylem tissue. Enrichment is expressed relative to the mean overlap observed in 1000 random permutations of H3K4me3 and H3K27me3 regions.

			H3K4me3				H3K27me3			
		**Total annotations**	**# Obs[a]**	**Obs (%)[b]**	**Enrichment**	***P*-value[c]**	**# Obs[a]**	**Obs (%)[b]**	**Enrichment**	***P*-value[c]**
Class I	LARD	106 420	6 153	5.8%	0.58	< 2.2e-16*	6 326	5.9%	1.14	< 2.2e-16*
	LINE	15 816	657	4.2%	0.35	< 2.2e-16*	431	2.7%	0.43	< 2.2e-16*
	LTR	68 051	2 515	3.7%	0.25	< 2.2e-16*	1 828	2.7%	0.35	< 2.2e-16*
	Unclassified	16 444	1 348	8.2%	0.79	4.10e-12*	967	5.9%	1.08	9.49e-02
	SINE	227	9	4.0%	0.56	2.16e-01	5	2.2%	0.60	5.75e-01
	TRIM	3 756	64	1.7%	0.19	< 2.2e-16*	107	2.8%	0.61	3.49e-05*
	VIPER/DIRS	940	53	5.6%	0.55	3.10e-04*	38	4.0%	0.76	2.30e-01
Class II	Unclassified	324	22	6.8%	0.74	3.82e-01	12	3.7%	0.77	6.95e-01
	Helitrons	15 176	1 545	10.2%	0.83	1.35e-08*	316	2.1%	0.32	< 2.2e-16*
	Maverick	581	31	5.3%	0.44	6.36e-05*	94	16.2%	2.60	7.31e-08*
	MITE	3 029	36	1.2%	0.15	< 2.2e-16*	73	2.4%	0.57	7.54e-05*
	TIR/CACTA	7 481	271	3.6%	0.34	< 2.2e-16*	259	3.5%	0.62	8.51e-10*

[a]Number of observed (Obs) annotations overlapping H3K4me3 or H3K27me3 peak regions

[b]Percentage of total annotations overlapping H3K4me3 or H3K27me3 peak regions

[c]Fisher's exact test. Significant results (Bonferroni-adjusted $P < 0.05$) are indicated by *.

Table S3. Common and unique *Arabidopsis thaliana* homologs of *E. grandis* developing xylem H3K27me3 targets with H3K27-trimethylated genes in various *A. thaliana* organs and tissues. DSX, developing secondary xylem (this study).

Dataset	Unique targets	Total targets	Unique proportion
Seedling[3–9]	5,224	13,237	0.395
DSX (*E. grandis*)	753	2,448	0.308
Root vascular cylinder[10]	110	761	0.145
Endosperm[11]	131	1,773	0.074
Leaf[12,13]	321	7,570	0.042
Shoot apical meristem[13]	218	6,071	0.036
Callus[12]	96	3,990	0.024

Table S4. Means, Kolmogorov-Smirnov test statistic values (D) and *P*-values for gene categories with different combinations of H3K4me3 and H3K27me3 (Fig. 4c).

Category	n (genes)	Mean*	K4me3$^+$ K27me3$^-$	K4me3$^+$ K27me3$^+$	K4me3$^-$ K27me3$^+$	K4me3$^-$ K27me3$^-$	DSX
**K4me3$^+$ K27me3$^-$**	16753	2.417	-	D = 0.56203	D = 0.71071	D = 0.50247	D = 0.15448
**K4me3$^+$ K27me3$^+$**	2580	1.762	*P* < 2.2e-16	-	D = 0.26259	D = 0.098123	D = 0.41227
**K4me3$^-$ K27me3$^+$**	2548	1.289	*P* < 2.2e-16	*P* < 2.2e-16	-	D = 0.22472	D = 0.58404
**K4me3$^-$ K27me3$^-$**	14121	1.632	*P* < 2.2e-16	*P* < 2.2e-16	*P* < 2.2e-16	-	D = 0.36412
**DSX**	27595	2.271	*P* < 2.2e-16	*P* < 2.2e-16	*P* < 2.2e-16	*P* < 2.2e-16	-

***Shannon entropy mean value**

Table S5. Post-hoc analysis of a Pearson's Chi-squared test comparing groups of genes with different histone methylation status according to their relative expression (Fig. 4d). Counts represent the number of genes for each tissue that showed a maximum relative expression in that tissue. The adjusted standardized residual values are the corresponding *z*-score; values greater than 2.32 or less than -2.32 indicate a one-tailed significance value of $P < 0.01$. DSX, developing secondary xylem; Fl, flowers; ML, mature leaves; Ph, phloem; Rt, roots; ST, shoot tips; YL, young leaves.

			Tissue							Total
			**DSX**	**Fl**	**ML**	**Ph**	**Rt**	**ST**	**YL**	
**Group**	**Bivalent**	**Count**	272	612	388	278	450	372	207	2579
		**% within Tissue**	1.6%	2.6%	3.1%	1.9%	4.9%	4.4%	1.5%	2.6%
		**Adjusted Residual**	-8.8	.4	3.9	-6.0	14.5	10.8	-9.2	
	**DSX-expressed**	**Count**	5451	5971	3443	4648	1702	2159	4209	27583
		**% within Tissue**	32.3%	25.6%	27.6%	31.2%	18.4%	25.3%	29.5%	27.7%
		**Adjusted Residual**	14.6	-8.0	-.3	10.3	-20.9	-5.1	5.3	
	**Genome**	**Count**	5451	8048	4133	4856	3315	2813	4819	33435
		**% within Tissue**	32.3%	34.5%	33.1%	32.6%	35.9%	33.0%	33.8%	33.6%
		**Adjusted Residual**	-3.9	3.6	-1.2	-2.8	5.0	-1.2	.6	
	**H3K27me3**	**Count**	399	1242	712	451	1187	685	448	5124
		**% within Tissue**	2.4%	5.3%	5.7%	3.0%	12.9%	8.0%	3.1%	5.1%
		**Adjusted Residual**	-18.0	1.5	3.0	-12.7	35.2	12.6	-11.7	
	**H3K4me3**	**Count**	3926	4108	2555	3561	1090	1375	2711	19326
		**% within Tissue**	23.2%	17.6%	20.5%	23.9%	11.8%	16.1%	19.0%	19.4%
		**Adjusted Residual**	13.8	-7.8	3.2	15.0	-19.4	-8.0	-1.3	
	**Unmodified**	**Count**	1398	3316	1254	1122	1488	1126	1868	11572
		**% within Tissue**	8.3%	14.2%	10.0%	7.5%	16.1%	13.2%	13.1%	11.6%
		**Adjusted Residual**	-14.9	14.2	-5.9	-16.9	14.2	4.8	6.0	
**Total**		**Count**	16897	23297	12485	14916	9232	8530	14262	99619
		**% within Tissue**	100.0%	100.0%	100.0%	100.0%	100.0%	100.0%	100.0%	100.0%

Table S6. Strand-specific RNA-seq library reads and mapping efficiencies to the *E. grandis* genome (v.1.1).

Tissue sample		Clean reads	Mapped reads	Mapping rate (%)	Concordance (%)
W7	Left reads	25,136,950	22,890,202	91.1%	83.7%
	Right reads	25,136,950	23,232,584	92.4%	
W8	Left reads	22,534,468	20,588,250	91.4%	84.1%
	Right reads	22,534,468	20,886,182	92.7%	

Table S7. Pearson correlation coefficient values between nett histone modification signals and log_2-transformed RNA-seq data for DSX tissue across annotated *E. grandis* genes (this study) and *A. thaliana* genes[8,14] for each of 40 bins. The transcription start site is represented by bin 21. The red and blue heat map indicates correlation strength for H3K4me3 and H3K27me3, respectively.

	H3K4me3		H3K27me3	
Bin	*E. grandis*	*A. thaliana*	*E. grandis*	*A. thaliana*
1	0.056	0.114	0.020	0.018
2	0.054	0.118	0.022	0.023
3	0.053	0.111	0.020	0.019
4	0.051	0.110	0.013	0.014
5	0.051	0.109	0.005	0.014
6	0.049	0.113	0.000	0.018
7	0.047	0.113	-0.003	0.024
8	0.046	0.109	-0.006	0.018
9	0.046	0.108	-0.010	0.020
10	0.043	0.110	-0.011	0.023
11	0.040	0.119	-0.013	0.030
12	0.036	0.129	-0.021	0.022
13	0.033	0.134	-0.028	0.028
14	0.031	0.148	-0.036	0.030
15	0.028	0.161	-0.046	0.033
16	0.026	0.165	-0.058	0.024
17	0.020	0.171	-0.076	0.024
18	0.017	0.177	-0.101	0.017
19	0.060	0.220	-0.142	-0.006
20	0.218	0.321	-0.194	-0.040
21	0.433	0.470	-0.209	-0.156
22	0.560	0.608	-0.188	-0.151
23	0.617	0.655	-0.157	-0.133
24	0.639	0.659	-0.132	-0.127
25	0.647	0.619	-0.117	-0.133
26	0.642	0.556	-0.114	-0.14
27	0.619	0.478	-0.118	-0.132
28	0.578	0.384	-0.121	-0.127
29	0.521	0.301	-0.125	-0.124
30	0.455	0.253	-0.128	-0.124
31	0.390	0.206	-0.131	-0.119
32	0.335	0.175	-0.134	-0.109
33	0.288	0.140	-0.133	-0.112
34	0.245	0.124	-0.130	-0.110
35	0.209	0.109	-0.127	-0.099
36	0.177	0.102	-0.121	-0.092
37	0.149	0.095	-0.116	-0.090
38	0.124	0.089	-0.111	-0.084
39	0.098	0.090	-0.103	-0.080
40	0.074	0.093	-0.097	-0.072

Table S8. Effect size of various significant H3K4me3 and H3K27me3 signal variables in predicting power-transformed FPKM values in *E. grandis* developing secondary xylem. Eta squared (η^2) values indicate the effect size that each variable uniquely contributes to the model. The bold variables indicate those used for the simplified model in Figure 5a.

Variable	Parameter estimate	Standard error	*t*-value	P-value	η^2
Intercept	3.27E-01	4.38E-03	74.61	<2.2×10-16	-
**H3K4me3_signal_bin25**	3.85E-03	4.61E-05	83.66	<2.2×10-16	0.2353
H3K4me3_peak_length	1.66E-04	5.80E-06	28.58	<2.2×10-16	0.0275
H3K27me3_peak_length	-1.11E-04	7.29E-06	-15.25	<2.2×10-16	0.0078
**H3K27me3_signal_bin21**	-1.84E-03	1.81E-04	-10.15	<2.2×10-16	0.0035
H3K4me3_total_signal	-8.99E-04	9.39E-05	-9.57	<2.2×10-16	0.0031
H3K27me3_total_signal	-2.25E-03	2.44E-04	-9.20	<2.2×10-16	0.0028

Table S9. Pearson correlation coefficient values between nett histone modification signals[8] and power-transformed RNA-seq data[14] for *A. thaliana* seedlings for each of 40 bins. The transcription start site is represented by bin 21. Red and blue shading indicates the strength of positive and negative correlations, respectively.

Bin	H3K9me2	H3K27me1	H3K27me3	H3K4me2	H3K4me3	H3K36me2	H3K36me3	H3K9ac	H3K18ac
1	0.065	0.052	0.018	0.135	0.114	0.129	0.136	0.134	0.094
2	0.071	0.057	0.023	0.136	0.118	0.132	0.139	0.141	0.093
3	0.069	0.055	0.019	0.136	0.111	0.132	0.135	0.138	0.089
4	0.065	0.051	0.014	0.135	0.110	0.131	0.138	0.136	0.086
5	0.065	0.049	0.014	0.134	0.109	0.133	0.135	0.138	0.085
6	0.072	0.050	0.018	0.139	0.113	0.137	0.141	0.140	0.088
7	0.069	0.051	0.024	0.140	0.113	0.134	0.142	0.143	0.092
8	0.067	0.050	0.018	0.139	0.109	0.132	0.141	0.143	0.087
9	0.064	0.052	0.020	0.139	0.108	0.123	0.145	0.142	0.087
10	0.070	0.055	0.023	0.139	0.110	0.127	0.145	0.144	0.090
11	0.081	0.069	0.030	0.148	0.119	0.132	0.148	0.152	0.095
12	0.081	0.066	0.022	0.155	0.129	0.132	0.151	0.160	0.095
13	0.078	0.063	0.028	0.151	0.134	0.123	0.149	0.161	0.097
14	0.085	0.073	0.030	0.156	0.148	0.126	0.158	0.169	0.100
15	0.091	0.078	0.033	0.157	0.161	0.130	0.164	0.180	0.111
16	0.085	0.068	0.024	0.150	0.165	0.119	0.166	0.184	0.103
17	0.094	0.080	0.024	0.148	0.171	0.122	0.175	0.187	0.101
18	0.107	0.093	0.017	0.136	0.177	0.125	0.189	0.184	0.096
19	0.126	0.112	-0.006	0.138	0.220	0.137	0.242	0.207	0.103
20	0.133	0.122	-0.040	0.165	0.321	0.146	0.346	0.276	0.126
21	0.031	0.002	-0.156	0.205	0.470	0.074	0.475	0.404	0.104
22	-0.031	-0.066	-0.151	0.289	0.608	0.054	0.573	0.546	0.158
23	-0.042	-0.071	-0.133	0.340	0.655	0.055	0.604	0.572	0.195
24	-0.036	-0.064	-0.127	0.357	0.659	0.070	0.599	0.545	0.200
25	-0.038	-0.062	-0.133	0.360	0.619	0.064	0.585	0.489	0.163
26	-0.049	-0.068	-0.140	0.356	0.556	0.060	0.579	0.431	0.124
27	-0.018	-0.033	-0.132	0.349	0.478	0.093	0.579	0.393	0.113
28	-0.015	-0.024	-0.127	0.326	0.384	0.101	0.567	0.349	0.091
29	-0.011	-0.025	-0.124	0.285	0.301	0.112	0.544	0.296	0.075
30	-0.006	-0.019	-0.124	0.264	0.253	0.138	0.519	0.268	0.073
31	-0.003	-0.009	-0.119	0.239	0.206	0.165	0.490	0.243	0.069
32	0.016	0.005	-0.109	0.220	0.175	0.192	0.459	0.222	0.071
33	0.004	-0.008	-0.112	0.195	0.140	0.206	0.419	0.197	0.060
34	0.012	-0.002	-0.110	0.183	0.124	0.234	0.385	0.181	0.064
35	0.022	0.008	-0.099	0.170	0.109	0.253	0.341	0.175	0.073
36	0.031	0.014	-0.092	0.159	0.102	0.261	0.309	0.168	0.076
37	0.033	0.016	-0.090	0.146	0.095	0.266	0.270	0.158	0.076
38	0.035	0.018	-0.084	0.137	0.089	0.265	0.243	0.150	0.079
39	0.032	0.018	-0.080	0.135	0.090	0.266	0.221	0.145	0.080
40	0.039	0.023	-0.072	0.132	0.093	0.267	0.207	0.150	0.089

Table S10. Effect size of nine histone modification signals[8] on the estimation of *A. thaliana* seedling RNA-seq data[14]. Eta squared (η^2) values indicate the effect size that each variable uniquely contributes to the model. Parameter estimates are based on a model trained on 13,261 genes and tested on 19,694 genes.

Variable	Parameter estimate	Standard error	*t* value	*P*-value	η^2
Intercept	5.82E-01	9.78E-03	59.54	<2.2×10-16	-
h3k4me3_signal_bin24	1.05E-01	2.16E-03	48.81	<2.2×10-16	0.0956
h3k9ac_signal_bin23	5.57E-02	1.85E-03	30.03	<2.2×10-16	0.0362
h3k18ac_signal_bin24	-7.51E-02	3.01E-03	-24.93	<2.2×10-16	0.0249
h3k27me1_signal_bin23	-7.84E-02	3.26E-03	-24.07	<2.2×10-16	0.0233
h3k36me2_signal_bin40	3.19E-02	2.05E-03	15.55	<2.2×10-16	0.0097
h3k4me2_signal_bin25	2.76E-02	1.93E-03	14.32	<2.2×10-16	0.0082
h3k36me3_signal_bin23	1.13E-02	1.11E-03	10.18	<2.2×10-16	0.0042
h3k27me3_signal_bin21	-1.60E-02	1.84E-03	-8.67	<2.2×10-16	0.0030
h3k9me2_signal_bin26	-1.77E-02	3.11E-03	-5.70	1.19E-08	0.0013

References

1. Hussey, S. G., Mizrachi, E., Groover, A., Berger, D. K. & Myburg, A. A. Genome-wide mapping of histone H3 lysine 4 trimethylation in *Eucalyptus grandis* developing xylem. *BMC Plant Biol.* **15,** 117 (2015).

2. Landt, S. G. *et al.* ChIP-seq guidelines and practices of the ENCODE and modENCODE consortia. *Genome Res.* **22,** 1813–1831 (2012).

3. Bouyer, D. *et al.* Polycomb Repressive Complex 2 controls the embryo-to-seedling phase transition. *PLoS Genet.* **7,** e1002014 (2011).

4. Charron, J.-B. F., He, H., Elling, A. A. & Deng, X. Dynamic landscapes of four histone modifications during deetiolation in Arabidopsis. *Plant Cell* **21,** 3732–3748 (2009).

5. Dong, X. *et al.* Natural variation of H3K27me3 distribution between two *Arabidopsis* accessions and its association with flanking transposable elements. *Genome Biol.* **13,** R117 (2012).

6. Farrona, S. *et al.* Tissue-specific expression of *FLOWERING LOCUS T* in *Arabidopsis* is maintained independently of polycomb group protein repression. *Plant Cell* **23,** 3204–3214 (2011).

7. Lu, F., Cui, X., Zhang, S., Jenuwein, T. & Cao, X. *Arabidopsis REF6* is a histone H3 lysine 27 demethylase. *Nat. Genet.* **43,** 715–721 (2011).

8. Luo, C. *et al.* Integrative analysis of chromatin states in *Arabidopsis* identified potential regulatory mechanisms for natural antisense transcript production. *Plant J.* **73,** 77–90 (2013).

9. Zhang, X. *et al.* Whole-genome analysis of histone H3 lysine 27 trimethylation in *Arabidopsis*. *PLoS Biol.* **5,** e129 (2007).

10. De Lucas, M. *et al.* Transcriptional regulation of *Arabidopsis* Polycomb Repressive Complex 2 coordinates cell type proliferation and differentiation. *Plant Cell Adv. Publ. Publ.* (2016). doi:10.1105/tpc.15.00744

11. Weinhofer, I., Hehenberger, E., Roszak, P., Hennig, L. & Köhler, C. H3K27me3 profiling of the endosperm implies exclusion of polycomb group protein targeting by DNA methylation. *PLoS Genet.* **6,** e1001152 (2010).

12. He, C., Chen, X., Huang, H. & Xu, L. Reprogramming of H3K27me3 is critical for acquisition of pluripotency from cultured *Arabidopsis* tissues. *PLoS Genet.* **8,** e1002911 (2012).

13. Lafos, M. *et al.* Dynamic regulation of H3K27 trimethylation during *Arabidopsis* differentiation. *PLoS Genet.* **7,** e1002040 (2011).

14. Liu, J. *et al.* *CURLY LEAF* regulates gene sets coordinating seed size and lipid biosynthesis in Arabidopsis. *Plant Physiol.* **171,** 424–436 (2016).
